# Potential biomarkers for late-onset and term preeclampsia: A scoping review

**DOI:** 10.3389/fphys.2023.1143543

**Published:** 2023-03-10

**Authors:** Luhao Han, Olivia J. Holland, Fabricio Da Silva Costa, Anthony V. Perkins

**Affiliations:** ^1^ School of Pharmacy and Medical Sciences, Griffith University, Gold Coast, QLD, Australia; ^2^ Maternal Fetal Medicine Unit, Gold Coast University Hospital, Gold Coast, QLD, Australia; ^3^ School of Medicine and Dentistry, Griffith University, Gold Coast, QLD, Australia; ^4^ School of Health, University of the Sunshine Coast, Sunshine Coast, QLD, Australia

**Keywords:** late-onset preeclampsia, term preeclampsia, biomarker, prediction model, screening

## Abstract

Preeclampsia is a progressive, multisystem pregnancy disorder. According to the time of onset or delivery, preeclampsia has been subclassified into early-onset (<34 weeks) and late-onset (≥34 weeks), or preterm (<37 weeks) and term (≥37 weeks). Preterm preeclampsia can be effectively predicted at 11–13 weeks well before onset, and its incidence can be reduced by preventively using low-dose aspirin. However, late-onset and term preeclampsia are more prevalent than early forms and still lack effective predictive and preventive measures. This scoping review aims to systematically identify the evidence of predictive biomarkers reported in late-onset and term preeclampsia. This study was conducted based on the guidance of the Joanna Briggs Institute (JBI) methodology for scoping reviews. The Preferred Reporting Items for Systematic Reviews and Meta-Analysis extension for scoping reviews (PRISMA-ScR) was used to guide the study. The following databases were searched for related studies: PubMed, Web of Science, Scopus, and ProQuest. Search terms contain “preeclampsia,” “late-onset,” “term,” “biomarker,” or “marker,” and other synonyms combined as appropriate using the Boolean operators “AND” and “OR.” The search was restricted to articles published in English from 2012 to August 2022. Publications were selected if study participants were pregnant women and biomarkers were detected in maternal blood or urine samples before late-onset or term preeclampsia diagnosis. The search retrieved 4,257 records, of which 125 studies were included in the final assessment. The results demonstrate that no single molecular biomarker presents sufficient clinical sensitivity and specificity for screening late-onset and term preeclampsia. Multivariable models combining maternal risk factors with biochemical and/or biophysical markers generate higher detection rates, but they need more effective biomarkers and validation data for clinical utility. This review proposes that further research into novel biomarkers for late-onset and term preeclampsia is warranted and important to find strategies to predict this complication. Other critical factors to help identify candidate markers should be considered, such as a consensus on defining preeclampsia subtypes, optimal testing time, and sample types.

## Introduction

Preeclampsia (PE) is a progressive, multisystem pregnancy disorder that can be a serious, even fatal condition. It affects 2%–4% of pregnancies worldwide and is responsible for nearly 46,000 maternal deaths and around 500,000 fetal and neonatal deaths every year (Magee et al., 2022b). PE not only leads to adverse health outcomes for mothers and babies but also produces a substantial financial burden on the healthcare system. The healthcare cost for pregnancies with PE is significantly higher than uncomplicated pregnancies, including higher inpatient costs, birth costs, and postpartum costs, especially for newborns admitted to Neonatal Intensive Care Unit (NICU) (Fox et al., 2017).

The etiology of PE is still not fully understood, but the understanding of this disorder has been greatly improved. Increasing evidence has shown that PE is a complex and heterogeneous disorder, as reflected in several aspects, such as pathophysiology, clinical phenotypes, screening effectiveness, aspirin prevention performance, and clinical outcomes. Previously, PE was diagnosed as a new onset of hypertension and proteinuria after 20 weeks of gestation. The diagnostic definition was broadened in 2018 by the International Society of the Study of Hypertension in Pregnancy (ISSHP). The latest ISSHP guideline (2021) characterizes PE as hypertension arising *de novo* plus one or more other conditions, including proteinuria, maternal organ dysfunctions, and uteroplacental dysfunction such as fetal growth restriction, abnormal umbilical artery Doppler, and imbalance of angiogenic markers (increased soluble fms-like tyrosine/placental growth factor (sFlt/PlGF) ratio or reduced PlGF) at or after 20 weeks (Magee et al., 2022a).

Previous research divided PE into early and late forms, whereas there is no consistent classification of PE subtypes. The gestational age of 34 or 37 is the cut-off value and is defined by the time of disease onset, diagnosis, or delivery. PE is widely accepted to be subclassified into early-onset (<34 weeks) and late-onset (≥34 weeks), or preterm (delivery <37 weeks) and term (delivery ≥37 weeks) (Poon et al., 2019). Despite the early and late subtypes sharing similar clinical symptoms, recent studies suggest that they have varied pathophysiology (Leavey et al., 2016; Wang et al., 2020; Ren et al., 2021). Moreover, subtyping PE based on etiology has been proposed (Roberts et al., 2021; Than et al., 2022).

A biomarker is an indicator of normal biological processes, pathogenic processes, or biological responses to an exposure or intervention that could be used to predict, diagnose, and monitor diseases (Cagney et al., 2018). This broad definition contains different types of biomarkers, such as molecular, histologic, radiographic, or physiologic characteristics. Useful biomarkers like biochemical markers (PlGF) or biophysical markers (mean arterial pressure, MAP) could improve the effectiveness of risk stratification for PE pregnancies, thereby creating a window of opportunity for clinicians to take preventative actions for high-risk women. Nevertheless, much previous research predicted PE as one type, and some focused more on reporting prediction achievement for the early form of PE. Apart from that, many biomarker studies tried to discover candidate predictors by using blood samples collected during PE diagnosis or placenta samples obtained after delivery, while those altered biomarkers may show better diagnostic value and reflect pathogenesis than the prediction.

The difference in detection rates for predicting PE subtypes presents another challenge. For example, the Fetal Medicine Foundation (FMF) developed a first-trimester screening model which combines maternal factors with biochemical markers (PlGF and pregnancy-associated plasma protein A, PAPP-A) and biophysical markers (uterine artery pulsatility index, UtA-PI, and MAP). This model achieves a high detection rate for predicting preterm PE (75%) at a 10% false positive rate (FPR), making PE screening clinically useful for this subtype. However, the detection rate for predicting term PE is less satisfactory (41%), and the biochemical markers did not improve in predicting term PE (Tan et al., 2018). This may indicate that the same prediction model and biomarkers are not suitable for the late type of PE. Further ongoing research should place a priority on improving prediction for late-onset/term PE since the rate of late type is substantially higher than the early type. The incidences of early-onset and late-onset PE are 30% and 70% in developing countries, as well as 10% and 90% in developed countries (Robillard et al., 2019).

A scoping review is a method of evidence synthesis that systematically identifies the evidence on a particular topic or field. In contrast to systematic reviews, scoping reviews address broader research questions and integrate heterogeneous evidence through a comprehensive search process. Considering the numerous and diverse biomarkers reported in PE prediction studies, this study intends to conduct a scoping review summarising the current state of knowledge of biomarkers for predicting late-onset and term PE. To reflect current challenges in PE prediction, the review will focus on molecular biomarkers tested in maternal blood or urine before late-onset and term PE diagnosis published within the past decade. It will provide an overview of current evidence on predictive biomarkers for late-onset and term PE and provide an in-depth analysis of screening for the late forms of PE. In a preliminary search, no current systematic reviews were found in the Cochrane Database of Systematic Reviews and JBI Evidence Synthesis website. No current or underway scoping reviews specifically addressing biomarkers for late-type PE were identified. The objective is to systematically analyse the recent literature in order to identify potentially useful biomarkers for predicting late-onset and term preeclampsia with the ultimate goal of improving the efficiency of PE screening.

## Methods

### Study design

This scoping review was conducted by the latest JBI methodology for scoping reviews (Peters et al., 2022). The checklist of Preferred Reporting Items for Systematic Reviews and Meta-Analysis extension for scoping reviews (PRISMA-ScR) was applied to report the review (Tricco et al., 2018). The review protocol was registered in Open Science Framework (Registration DOI https://doi.org/10.17605/OSF.IO/XW9QU).

### Research questions

The following research question and selection criteria were defined by the PCC framework (“Participants,” “Concept,” “Context”).

Primary question: Is there any effective molecular biomarker reported in the previous literature that could potentially predict late-onset and term PE?

Secondary questions:

• Which gestations that significantly changed molecular biomarkers have been detected?
• What techniques have been used to study molecular biomarkers?

### Eligibility criteria

Publications were chosen if study participants were pregnant women, and molecular biomarkers (including proteins, nucleic acids, and metabolites) for late-onset or term PE were detected. Late-onset and term PE are defined according to disease onset, diagnosis, or delivery gestation ≥34 and 37, respectively. Study sample types are limited to maternal blood, serum, plasma, and urine. Only full-text articles published in English from 2012 to August 2022 were included. At the full-text screening stage, the number of potential biomarkers was considerable. Therefore, the criteria were refined to include biomarkers tested before diagnosing PE or clinical symptoms manifest for predictive purposes.

### Search strategy

The following four databases: PubMed, Web of Science, Scopus, and ProQuest, were searched for relevant studies. Search terms contain “preeclampsia,” “late-onset,” “term,” “biomarker,” or “marker,” and other synonyms combined as appropriate using the Boolean operators “AND” and “OR”. Studies identified were limited to those published in English from 2012 to August 2022. More details, such as the electronic search strategy and keywords, can be found in the protocol (DOI https://doi.org/10.17605/OSF.IO/XW9QU).

### Data extraction and synthesis

Covidence, a reference manager for screening and data extraction, was used to select published studies for inclusion. All search records were imported into Covidence, and duplicates were identified and removed automatically and manually. One reviewer (LH) screened the titles and abstracts. Two reviewers (LH and OH) independently conducted the full-text screening. Disagreements were resolved through discussion with the third and fourth reviewers (AP and FD). Data were extracted by one author (LH), including publication information, method, and results of biomarkers, and then confirmed by other reviewers (OH, AP and FD).

## Results

### Search results

**FIGURE 1:**
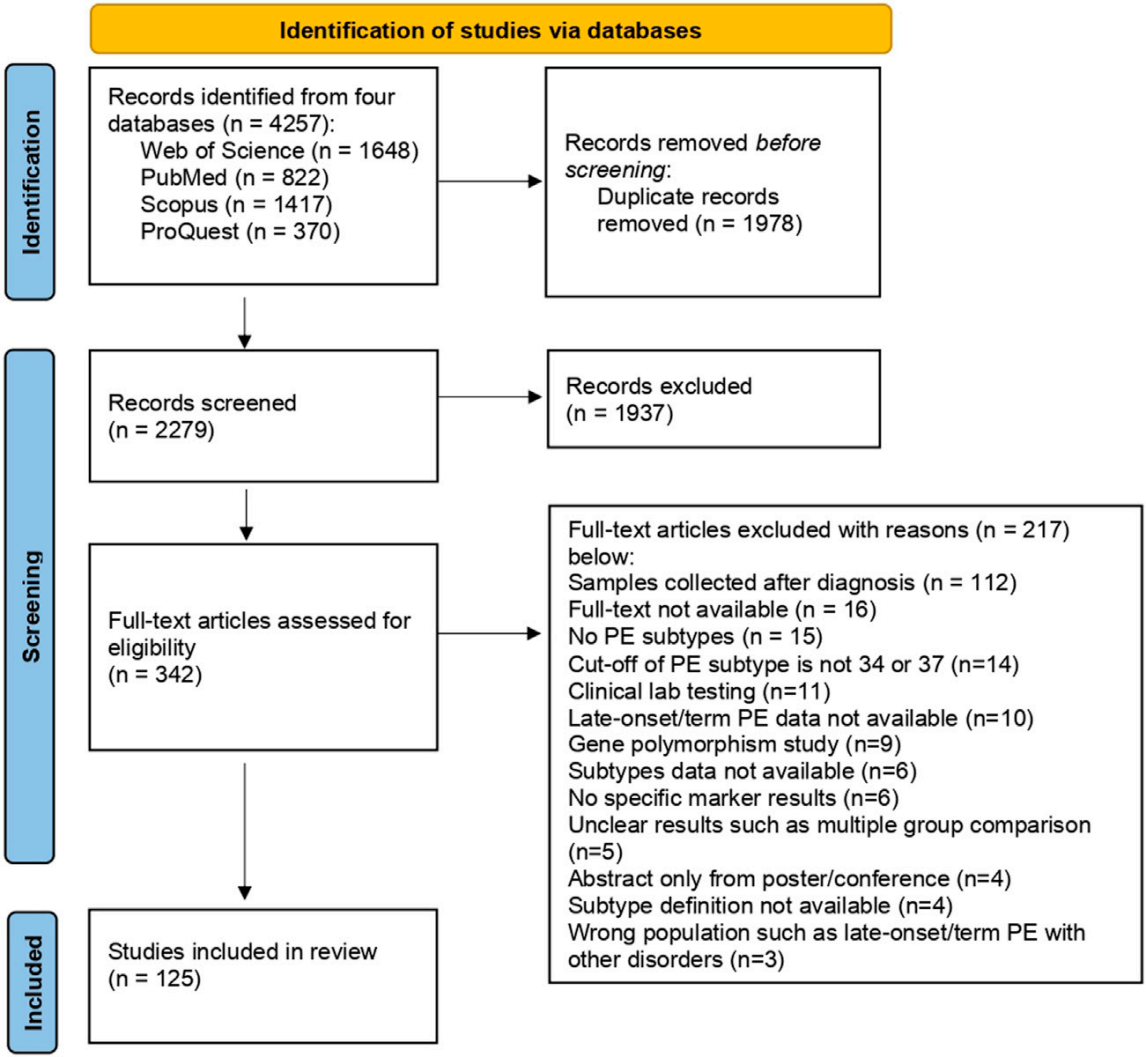
PRISMA flow diagram of the study selection process.

A flow diagram (Figure 1) shows the study selection and screening process following the PRISMA guidelines (Page et al., 2021). A total of 4,257 records were found after a systematic search. After removing 1,978 duplicates, 2,279 studies were screened by abstract, and 125 articles with full text were included in the final assessment. Based on our analysis of those included studies, there were two ways to study molecule biomarkers for predicting late-onset and term PE. The first (66/125, 53%) is to study molecular biomarkers alone, such as reporting level change and association with disease. The other (59/125, 47%) investigated molecular biomarkers with a combination of other predictors, such as maternal risk factors and biophysical markers, to build multivariable models.

Sixty-six studies investigated biomarkers alone (summarized in Supplementary Table S1 **)**. The majority are protein markers (study number = 48), and others are nucleic acids markers (study number = 9) and metabolic markers (study number = 9). The type of molecular markers varies, including protein, cell-free DNA, mitochondrial DNA, mRNA, microRNA, and metabolites. The most frequently studied biomarkers are proteins, especially angiogenesis factors, PlGF and sFlt-1, and placenta-expressed proteins, such as PAPP-A. Enzyme-linked immunosorbent assay (ELISA) and biochemical analyzer are the most popular methods for protein measurement, and reverse transcription polymerase chain reaction (RT-PCR) is the main approach for mRNA and microRNA study. Nuclear magnetic resonance (NMR) spectroscopy and liquid chromatography-mass spectrometry (LC-MS) are two primary techniques for metabolomics study. All protein markers were measured in serum and plasma samples. Five mRNA and microRNA studies used whole blood, and peripheral blood mononuclear cells (PBMC), and only one study analyzed urine samples.

**FIGURE 2:**
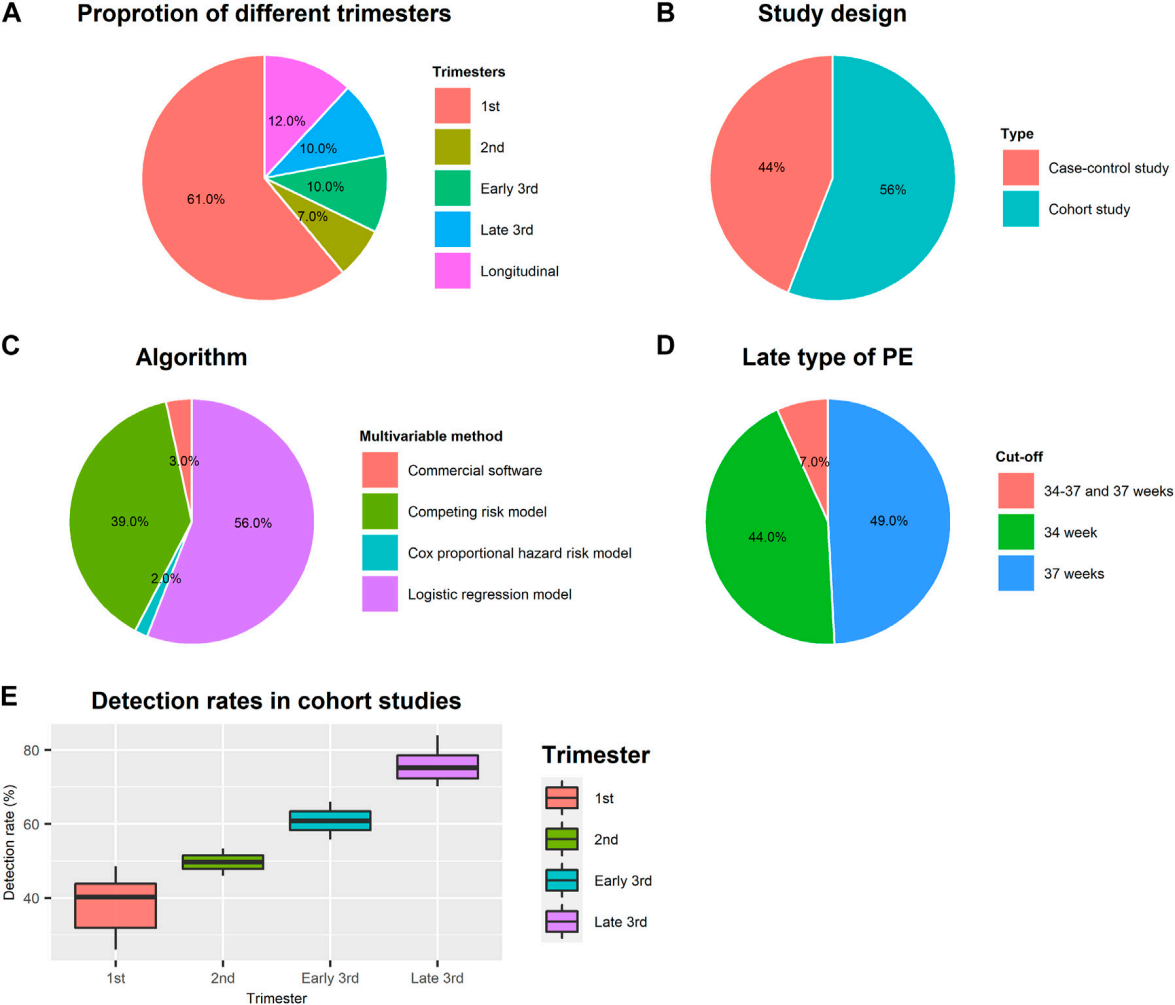
Study characteristics of multivariable model studies. **(A)** Proportion of multivariable model studies conducted in different trimesters; **(B)** Proportion of study design; **(C)** Proportion of algorithms used for multivariable model-building; **(D)** Cut-off value of gestational age for defining late type of PE; **(E)** Average detection rates in different gestation age in cohort studies.

Fifty-nine multivariable model studies combine maternal factors with biochemical and/or biophysical markers to predict late-onset/term PE. Table 1 lists seven longitudinal research studies, and Supplementary Table S2 summarizes the other studies focused on single trimester. Figure 2 displays an overview of the main characteristics of multivariable model studies. As shown in Figure 2A, most studies (61%) were conducted in the first trimester, and 12% were longitudinal studies. Only 7% and 20% were second and third trimester screening models. The study design includes 56% cohort and 44% case-control studies **(** Figure 2B). Figure 2C shows the type of algorithms used in building multivariable models, which are logistic regression (56%), competing risk models developed by FMF (39%), commercial software (3%), and Cox proportional hazard risk model (2%). According to searching results, reporting PE subtypes’ cut-off value varies among studies. Cut-offs of 30, 32, 34, and 37 weeks of gestational age were used to classify late-onset and term PE. Fourteen studies were excluded at the full-text screening stage because the cut-off is 30 or 32 (Figure 1). 49% reported PE ≥ 37 weeks, 44% were PE ≥ 34 weeks, and 7% separated late-type PE into 34–37 weeks and 37 weeks (Figure 2D).

**TABLE 1:** Summary of longitudinal studies that combined maternal factors with biomarkers.

Study	Country	PE subtypes	Population	Study design	Sample size	Biochemical marker	Algorithm	Screen GA (weeks)	Combined model	DR at 10%FPR	AUC	Note
Tarca et al. (2022)	USA	Term PE with delivery ≥37 weeks	A retrospective analysis of data from 1,150 pregnancies, previously described as part of a case-cohort	Longitudinal case-cohort	1,150	PlGF, sVEGR-1, sEng	Logistic regression	8–15	MF + MAP + PlGF + sVEGFR-1+sEng	36%	0.780	Sensitivity for term PE improved after 32 weeks; models performed similarly to the FMF algorithm when the same biomarker data were used.
								16–19		36%	0.710	
								20–23		41%	0.730	
								24–27		43%	0.770	
								28–31		39%	0.750	
								32–36		51%	0.820	
Andrietti et al. (2017)	UK	Term PE with delivery ≥37 weeks	From prospective screening for adverse obstetric outcomes in women attending routine second and third trimester visits in the UK, Dec 2010 - Aug 2014	Longitudinal prospective cohort		PlGF	Competing risks model	11–13	MF alone	40.5%	0.796	Measurements of UtA-PI, MAP, and PlGF in the first and/or second trimesters have a small or no effect on improving the prediction of PE in the early third trimester.
								11–13	MF + PlGF	42.8%	0.771	
								19–24		40.6%	0.750	
								30–34		55.8%	0.835	
Bredaki et al. (2016)	UK	Term PE with delivery ≥37 weeks	From prospective screening for adverse obstetric outcomes in women attending three routine visits in the UK, Mar 2006 - Apr 2014	Longitudinal prospective cohort	17,071	AFP	Competing risks model	11–13	MF alone	37%	0.721	Measuring serum AFP at 11–13 weeks is not a good predictive marker of PE.
								11–13	MF + AFP	37%	0.754	
					8,583			19–24		38%	0.770	
					8,609			30–34		NA	NA	
Wright et al. (2016a)	UK	Term PE with delivery ≥37 weeks	Prospective screening for adverse obstetric outcomes in women attending three routine hospital visits in the UK	Longitudinal prospective cohort	94,989	PAPP-A	Competing risk model	11–13	MF alone	37%	0.749	Measuring serum PAPP-A and β-hCG did not help predict term PE in the first and second trimesters. DR is slightly improved from 37% (MF only) to 45% at 30–34 weeks.
								11–13	MF + PAPP-A	38%	0.753	
								11–13	MF+ β-hCG	37%	0.748	
					7,597	β-HCG		19–24	MF+ β-hCG	38%	0.727	
					8,088	PAPP-A, β-hCG		30–34	MF + PAPP-A+β-hCG	45%	0.749	
Tsiakkas et al. (2016a)	UK	Term PE with delivery ≥37 weeks	Prospective screening for adverse obstetric outcomes in women attending three routine hospital visits in the UK, Mar 2006 - Dec 2014	Longitudinal prospective cohort	40,212	PlGF	Competing risk model	11–13	MF alone	37%	0.748	Compared to applying maternal factors only (37% at 10% FPR), combining with PLGF could modestly improve the detection rate from 30–34 weeks.
								11–13	MF + PLGF	40%	0.765	
					10,282			19–24		37%	0.757	
					10,400			30–34		54%	0.831	
					4,043			35–37		64%	0.874	
Tsiakkas et al. (2016b)	UK	Term PE with delivery ≥37 weeks	Prospective screening for adverse obstetric outcomes in women attending three routine hospital visits in the UK, Nov 2011 - Dec 2014	Longitudinal prospective cohort	7,066	sFlt-1	Competing risk model	11–13	MF alone	37%	0.748	The combined model with sFlt-1 improved the prediction of term PE at 30–34 and 35–37 weeks.
								11–13	MF + sFlt-1	37%	0.748	
					8,079			19–24		37%	0.748	
					8,472			30–34		52%	0.818	
					4,043			35–37		69%	0.896	
Wright et al. (2016b)	UK	Term PE with delivery ≥37 weeks	Prospective screening for adverse obstetric outcomes in women attending routine hospital visits in the UK, Nov 2011 -Jul 2014	Longitudinal prospective cohort	7,565	sFlt-1	Competing risk model		MF alone	41%	0.750	Screening sFlt-1 at 19–24 improves predicting PE at 30–34.
								19–24	MF + sFlt-1	41%	0.750	
					8,264			30–34		54%	0.825	
								Combined 19–24 and 30–34		64%	0.860	

PE, preeclampsia; GA, gestational age; DR, detection rate; FPR, false positive rate; AUC, area under the ROC curve; PlGF, placental growth factor; sVEGF-R1, soluble vascular endothelial growth factor receptor-1; sEng, soluble Endoglin; MF, maternal factor; MAP, mean arterial pressure; FMF, fetal medicine foundation; UtA-PI, Uterine artery pulsatility index; AFP, alpha-fetoprotein; PAPP-A, pregnancy-associated plasma protein A; β-hCG, beta human chorionic gonadotropin; sFlt-1, soluble fms-like tyrosine kinase-1.

As shown in Table 1, the detection rates at 10% FPR for screening term PE by maternal risk factors alone range from 36% to 41% at 11–13 weeks. Combined maternal factors with angiogenic markers (PlGF, sFlt-1, sEng) at 30–34 weeks are useful for screening term PE by identifying over 50% of term PE. We calculated the average detection rates in 52 studies conducted only in one trimester to compare the prediction performance difference. Case-control studies present a higher detection rate (mean = 51%, SD = 19%) than cohort studies (mean = 39% and SD = 7%) in the first trimester but with greater variance. In cohort studies, the mean detection rates in the first and second trimesters are 39% (SD = 7%) and 50% (SD = 5%) but increase to 61% (SD = 7%), and 76% (SD = 5%) in the early and late third trimesters (Figure 2E).

## Discussion

In this scoping review, we explored past research about predictive biomarkers for the late subtype of PE. One hundred twenty-five articles reported individual molecular biomarkers and biomarkers combined with maternal factors and/or biophysical markers. Together these results provide important insights into the possible ways of predicting the late form of PE. To become clinically useful, a potential biomarker should demonstrate clinical reproducibility, validity, and utility (Cagney et al., 2018; Ou et al., 2021). Evidence sufficient to qualify a biomarker depends on factors such as the context of use, potential benefits, and risks associated with its use (Sauer and Porter, 2021). Our results show that current molecular biomarker research for late PE remains at the discovery and validation stage. There is still a wide gap in translating biomarkers into clinical use. Further investigation is necessary to confirm the changed biomarkers and evaluate their efficiency in conjunction with other predictive measures.

## Predictive molecular markers

### Protein markers

Angiogenic and antiangiogenic factors have been widely investigated as biomarkers for PE for decades. PlGF and its soluble receptor sFlt-1, and sFlt-1/PlGF ratio, are the best clinically available markers for now and are recommended in commercial tests for preterm PE screening and diagnosis (Kmietowicz, 2022). The challenge for preterm PE now is clinical implementation and cost-effectiveness of tests. In this review, PlGF and sFlt-1 are also the most frequently studied biomarkers for late-onset and term PE, including 16 studies alone (Supplementary Table S1) and 50 studies combined with other predictors (Table 1; Supplementary Table S2). According to two longitudinal studies, multivariable models combining maternal factors with PlGF and sFlt-1 individually achieved detection rates (DR) of 54% and 52% at a 10% FPR at 30–34 weeks, which are higher than with maternal factors alone (DR = 37% at 10% FPR) (Tsiakkas et al., 2016a; Tsiakkas et al., 2016b). Moreover, soluble Endoglin (sEng) is another promising angiogenic marker that could identify half of late-onset PE cases at 30–33 weeks when combined with maternal factors (Lai et al., 2013b).

Another source of promising biomarkers is placenta-derived proteins. Placenta dysfunction is considered the main factor of PE development and leads to changes in the release of small molecules into the maternal circulation. Researchers applying fetal chromosomal anomalies screening biomarkers (β-hCG and PAPP-A) to predict PE found that the two markers were less useful for term PE compared to preterm PE (Scazzocchio et al., 2013; Teixeira et al., 2014; Wright et al., 2016a; Scazzocchio et al., 2017). Activin-A and inhibin-A are glycoprotein hormones expressed by the placenta and released into the maternal circulation. In two previous studies, activin-A increased at 30–33 and 36 weeks in late-onset PE and term PE, but not at 11–13 weeks, and the detection rate at 30–33 weeks was 36% at 10% FPR and increased to 50% when combined with maternal factors (Lai et al., 2013a; Wong et al., 2022). Another study proposed that inhibin-A is a better predictor than PlGF for late-onset PE at 12–14 weeks (Keikkala et al., 2021). Other placenta-related biomarkers such as HtrA3 (Wang et al., 2018), A disintegrin and metalloproteinase 12 (ADAM12) (Andres et al., 2022), growth differentiation factor 15 (GDF-15) (Cruickshank et al., 2021), tissue factor pathway inhibitor (TFPI) (MacDonald et al., 2021), and SPINT2 (Murphy et al., 2021) are also reported to change in the maternal circulation of late PE cases.

### Nucleic acid markers

Besides proteins, plasma and serum samples contain cell-free DNA (cfDNA) and RNA fragments. Fetal cfDNA testing is widely used for fetal aneuploidy screening and may be helpful for PE. Rolnik et al. found that total cfDNA is only increased in early-onset PE, not late-onset PE. Despite a significant decrease in the median fetal fraction in late-onset PE at 20–24 weeks, its capacity as a marker requires further validation (Rolnik et al., 2015). A recent study reported that cell-free RNA changes could predict the risk of PE before the onset of symptoms but did not specifically address subtypes of PE (Moufarrej et al., 2022).

Busnelli et al. reported that mitochondrial DNA copy number in maternal peripheral blood was lower in late-onset PE in the first trimester. However, this change is more associated with cases with Intrauterine growth restriction (IUGR) (Busnelli et al., 2019). Two studies investigated mRNA in maternal whole blood samples. One article reported that adrenomedullin (ADM) mRNA significantly decreased in maternal circulation up to 10–12 weeks before term PE onset (Whigham et al., 2019). Another study validated several genes in a test cohort based on bioinformatic analysis of GEO databases and identified three genes (*HDC, MS4A2, SLC18A2*) differentially expressed in late-onset PE (Lin et al., 2022). A few microRNA studies were performed to identify candidate biomarkers associated with late-onset PE and term PE (Winger et al., 2015; Mavreli et al., 2020; Whigham et al., 2020).

### Metabolomic markers

Nine studies reported that metabolic markers are significantly changed in maternal serum or plasma, and five multivariable model studies combined metabolic markers with maternal factors and/or other markers. Bahado-Singh et al. reported that the levels of 17 metabolites were significantly altered in late-onset PE cases at 11–13 weeks, and combining those differential metabolites with maternal factors achieved 76.6% sensitivity at 100% specificity in predicting late-onset PE (Bahado-Singh et al., 2013). Later, a study reported that a multivariable model (maternal weight plus UtA-PI and pyruvate, carnitine) yielded a 34.8% detection rate at 17.4% FPR in late-onset PE (Bahado-Singh et al., 2017). Another study found that stearoylcarnitine could modestly improve the combined model consisting of prior risk, MAP, PAPP-A, and PlGF and increase the detection rate from 27% to 32% for late-onset PE (Koster et al., 2015). Kuc et al. (2014) reported that glycylglycine was significantly changed in late-onset PE in the first trimester but did not improve the prediction model (prior risk combined with MAP). Through an untargeted metabolomics analysis in term PE cases, Sovio et al. (2020) identified 100 differential metabolites at 20/28 weeks and validated 33 of them at 36 weeks. 4-hydroxyglutamate and C-glycosyltryptophan showed independent predictive value for term PE.

Although metabolic markers could be potentially applied in prenatal screening, they have poor reproducibility because they are easily influenced by external factors such as diet, lifestyle, and the environment (Monni et al., 2021). Hence, fewer metabolic markers are validated among those studies. Compared to protein markers, data about the prediction efficacy of metabolic markers for the late form of PE is limited.

## Combined model screening

In clinical practice, the widely used approach to identify women with a high risk of PE is based on maternal risk factors defined by the American College of Obstetricians and Gynecologists (Gestational hypertension and preeclampsia: ACOG practice bulletin, number 222, 2020) and the National Institute for Health and Care Excellence (NICE, 2019) guidelines. However, the detection rates are low (41% and 34% for preterm and term PE at a 10% FPR). Various multivariable models that combined maternal risk factors with various markers have been developed to improve the PE prediction performance. The FMF first trimester prediction model successfully predicted early-onset and preterm PE with 90% and 75% detection rates at a 10% FPR (O'Gorman et al., 2016). In our study, 59 multivariable model studies reported data on prediction performance for late-onset or term PE (Table 1; Supplementary Table S2). Most of those studies (61%) were conducted in the first trimester, potentially due to the convenience of collecting blood samples. Most pregnant women have their first blood test for fetal chromosomal anomalies screening at 8–12 weeks, making the first trimester an accessible time to recruit pregnant participants and collect blood samples.

Nevertheless, the first-trimester screening may not be suitable for late-onset and term PE. We analyzed detection rates in cohort studies and found the average detection rate of multivariable models in the first trimester is 39% which is similar to using maternal risk factors alone. As shown in Figure 2E, the detection rates are improved with screening at increasing gestation age. A combination of maternal factors and biomarkers at 35–37 weeks’ gestation could identify about 76% term PE. This evidence suggests that the optimal time for biomarker screening may be later than the first trimester. However, data from the combined model used in late PE in the second and third trimesters are insufficient. Only 7% of multivariable model studies reported combined screening models in the second trimester, and 20% reported combined screening models in the third trimester.

## Considerations in biomarker studies for late-onset and term PE

### Subtype definition

PE involves multifactorial etiology and progresses dynamically during pregnancy. It can occur at various gestational ages, even postpartum, and display different grades of severity. Increasing publications encourage researchers to examine PE as a heterogeneous syndrome with subtypes instead of merging into one category (Roberts et al., 2021; Than et al., 2022). There is currently no consistent approach to classify PE subtypes. In this review, we only targeted late-onset and term PE, defined as after 34 and 37 weeks of gestation according to International Federation of Gynecology and Obstetrics (FIGO) guidelines (Poon et al., 2019). The cut-off value (34/37 weeks) is based on the gestation age of disease onset, diagnosis, or delivery. Nevertheless, this classification is limited since the timing of PE onset, diagnosis, and delivery are different, making the prediction results in each study less comparable. While the onset of PE may more accurately reflect disease pathophysiology, delivery time is likely to be related to disease severity, requiring delivery due to maternal and/or fetal symptoms, and possibly varied between healthcare institutions (Dimitriadis et al., 2023). Our analysis of PE subtype definitions in multivariable model studies found that 49% used ≥37 weeks and 44% used ≥34 weeks. 7% of those studies separated late PE into 34–37 weeks and ≥37 weeks. Questions remain about whether a cut-off of 34 or 37 weeks should be utilized and how to define the cut-off time.

ISSHP guidelines suggest PE should not be classified as “mild” or “severe” in an ongoing pregnancy. However, Villa et al. show that sFlt-1 concentration differs between severe and non-severe late-onset PE, which may indicate biomarker level change is related to the disease severity (Villa et al., 2013). Furthermore, researchers recently suggested that PE may be subtyped more accurately based on pathogenesis instead of gestational age at onset or delivery (Than et al., 2022). How we report and categorize PE subtypes is still up for debate and requires further research. Here, we underline the importance and need for consensus on classifying PE in future studies. Consistent definitions for PE subtypes will directly impact how researchers report the results and biomarker performance.

### Timing of biomarker test

Given that PE may involve multiple etiologies, it is unlikely that a single strategy could be used to predict all PE cases. One hypothesis proposes that multiple pathogenetic pathways may result in the same pathologic endpoint, placental dysfunction (Redman et al., 2022). The onset time and severity of placental dysfunction may vary in PE subtypes. In the early type, abnormal placentation probably occurs during early gestation and is the leading cause of pathology. However, for late PE, both placenta and maternal dysfunction, such as maternal cardiac dysfunction (Thilaganathan, 2020), may contribute to disease and progress later during pregnancy. The subclinical period in which the biomarkers begin to present significant change before diagnosis may create a window of opportunity for prediction and prevention. So far, very little is currently known about the subclinical period, which is critical to biomarker tests in the late type of PE.

It is hypothesized that the closer the disease onset, the more significant potential biomarkers change. Lai et al. (2013a, 2013b) measured activin-A and sEng at 11–13 weeks and 30–33 weeks and found these two proteins only present significant change at 30–33 weeks. Researchers from Australia reported a group of placental-derived proteins which are significantly changed in 36 weeks preceding term PE diagnosis from two cohort studies (Cruickshank et al., 2021; MacDonald et al., 2021; Murphy et al., 2021; Andres et al., 2022; Kandel et al., 2022; Wong et al., 2022). Other evidence also shows that gene expression in circulating neutrophils altered 8 weeks before term PE onset (Walsh et al., 2021). Moreover, as mentioned before, PlGF and sFlt-1 started improving the combined screening model at 30–34 weeks. Earlier identification of high-risk pregnancies allows interventive actions. Therefore, exploring the time threshold of biomarkers starting to show significant alteration in late-onset/term PE samples is likely to be a fruitful area of future research, as well as determining the optimal time point of screening benefit using biomarkers.

### Other factors

Other factors such as study design, sample type, and statistical method are critical for discovering and identifying potential biomarkers. Many PE studies have detected biomarkers in blood samples collected during PE diagnosis or in placenta samples obtained after delivery. However, this type of biomarker may be less capable of reflecting the change before the disease manifestations. In this review, we excluded these studies (*n* = 112, Figure 1) and limited the inclusion criteria to biomarkers tested prior to disease diagnosis. Also, we found that the average detection rate of case-control studies with the multivariable model is higher than cohort studies but with a greater standard deviation. The population in a cohort considers the disease frequency a key factor and may better reflect the actual prediction performance.

The current multivariable models are mainly based on logistic regression analysis (56%) and FMF-developed competing risk models (39%). Methods from machine learning algorithms or artificial intelligence for building prediction models have rapidly increased in popularity. A study that applied machine learning to combine maternal factors and clinical laboratory data could effectively predict late-onset PE from the second trimester to 34 weeks (Jhee et al., 2019). Another article utilized artificial intelligence and machine learning methods to evaluate the accuracy of prediction PE, although they only reported data on all PE cases and preterm PE (Ansbacher-Feldman et al., 2022). Machine learning seems to be a powerful tool to improve late-onset and term PE risk assessment by integrating large datasets, including clinical information, ultrasound imaging data, and biomarker tests. Building this type of model requires research and clinical data, which need close collaboration between researchers and clinicians. Additionally, expertise from multiple disciplines, such as data science, biomedical science, and clinical research will be critical.

## Strengths and limitations

In this review, heterogeneity and quality assessment have not been conducted to determine the clinical performance of biomarkers and prediction models. Additionally, heterogeneity among the study populations was not specifically accounted for. Therefore, detection rates were only roughly estimated for the combined model in cohort studies. In addition, this review mainly focuses on searching for potential prediction methods for late-onset and term PE and therefore does not summarize the data for early-onset/preterm and compare subtypes. Researching and comparing the differences between subtypes of PE is likely to be a worthwhile area of investigation because this may help to determine the mechanism behind PE subtypes. Our strength is that we specifically address the problem that there is scant attention in the research of late-onset/term PE and characterization of subtypes is essential for better prediction of late-onset/term PE.

## Conclusion

This study summarized the current predictive biomarkers for late-onset and term PE. Findings emphasize the necessity for further validation and optimization of prediction strategy for late PE through integrating new biomarkers and algorithms.

For better prediction of late-onset and term PE, we suggest that future studies should 1) use a consistent definition of subtypes PE and stratify cases into subtypes; 2) consider the time when biomarkers start to show a significant change in maternal samples; and 3) engage collaboration with clinicians to obtain more clinical information and data. Further, PE risk assessment may incorporate machine learning into the risk assessment system for monitoring disease progression and adverse outcomes.

## Data Availability

Publicly available datasets were analyzed in this study. This data can be found here: https://doi.org/10.17605/OSF.IO/XW9QU.
