# Supplementary material for: Potential biomarkers for late-onset and term preeclampsia: A scoping review
Source: Front Physiol. 2023 Mar 10;14:1143543. doi: 10.3389/fphys.2023.1143543 (PMC10036383; doi:10.3389/fphys.2023.1143543)
Supplement: Supplementary file 1 [file Table1.DOCX]

**Table s1. Summary of reported potential molecular biomarkers for predicting late-onset and term PE**

| **Biomarker type** | **Biomarker** | **PE subtypes** | **GA of testing (weeks)** | **Sample type** | **Method** | **Discriminative performance** | **Reference** |  |
| --- | --- | --- | --- | --- | --- | --- | --- | --- |
| Angiogenic and antiangiogenic protein | Placental growth factor, PlGF | LOPE and term PE | 11-14 | Serum | Not available | Significantly decreased in both late types | (Romero Infante et al., 2022) |  |
|  |  | LOPE | 11-20  Time of diagnosis | Serum | ELISA | Significantly decreased.  Group 1- SHS: AUC = 0.79, 53.6% sensitivity at 89.5% specificity; Group 2 OHS: AUC = 0.68, 93.3% sensitivity at 62.6% specificity | (Anto et al., 2021) |  |
|  |  | PE at 35-37 weeks and >37 weeks | Longitudinal | Serum | Biochemical analyzer | Lower PlGF levels (P < 0.001) in PE at 35-37 weeks; this association was not seen in PE > 37 weeks | (Fillion et al., 2020) |  |
|  |  | LOPE | 32-37 | Serum | Biochemical analyzer | AUC=0.94, 84.4% sensitivity at 90.4% specificity | (Birdir et al., 2018) |  |
|  |  | LOPE | 9-15  20-42 | Serum | Biochemical analyzer | Decreased at 9 -15 weeks | (Jaaskelainen et al., 2018) |  |
|  |  | LOPE | 11-13 | Serum | ELISA | No significant change | (Nuriyeva et al., 2017) |  |
|  |  | LOPE | 8-14  20-34 | Serum | Biochemical analyzer | AUC=0.679, 67% sensitivity at 68.8% specificity | (Andersen et al., 2016) |  |
|  |  | Term PE | Longitudinal | Serum | Biochemical analyzer | PlGF levels were significantly lower from 13 weeks onward | (Khalil et al., 2016) |  |
|  |  | LOPE | 20-41 | Serum | Biochemical analyzer | Significantly decreased | (Delic et al., 2014) |  |
|  |  | LOPE | 11-13  20-24  30-34 | Serum | Biochemical analyzer and ELISA | PlGF isoforms 1 were significantly lower at 30-34 weeks; PlGF isoforms 2 were significantly lower at three-time points | (Nucci et al., 2014) |  |
|  |  | LOPE | 12-14  18-20  26-28 | Serum | Biochemical analyzer | No significant change | (Villa et al., 2013) |  |
|  |  | PE at 34-37 weeks and >37 weeks | 20, 24, 28, 32, 36 | Serum | Biochemical analyzer | Significantly changed from 24 weeks in 34-37 weeks PE and 28 weeks in > 37 weeks PE | (Leanos-Miranda et al., 2012) |  |
|  |  | Term PE | Longitudinal | Serum | ELISA | A low level of PlGF was associated with a higher risk for term PE with SGA but no association of term PE without SGA | (Vatten et al., 2012) |  |
| Angiogenic and antiangiogenic protein | Soluble fms-like tyrosine-1, sFlt-1 | LOPE | 11 - 20  Time of diagnosis | Serum | ELISA | Significantly increased  Group1-SHS: AUC = 0.77, 55.7% sensitivity at 88.4% specificity  Group2-OHS: AUC = 0.76, 60.0% sensitivity at 88.4% specificity | (Anto et al., 2021) |  |
|  |  | PE at 35-37 and > 37 weeks | Longitudinal | Serum | Biochemical analyzer | Higher sFlt-1 levels (P < 0.001) in 35-37 weeks PE; this association was not seen in > 37 weeks PE. | (Fillion et al., 2020) |  |
|  |  | LOPE | 32-37 | Serum | Biochemical analyzer | AUC=0.89, 84.4% at 92% specificity | (Birdir et al., 2018) |  |
|  |  | LOPE | 9 - 15  20 - 42 | Serum | Biochemical analyzer | No significant change at 9 -15 weeks but increased 20-42 weeks | (Jaaskelainen et al., 2018) |  |
|  |  | Term PE | 11-14  22-24  32-36 | Plasma | ELISA | Significantly decreased only at 32- 36 weeks | (Palmer et al., 2017) |  |
|  |  | Term PE | Longitudinal | Serum | Biochemical analyzer | sFlt-1 levels did not differ significantly (P=0.044) | (Khalil et al., 2016) |  |
|  |  | LOPE | 20-41 | Serum | Biochemical analyzer | Significantly increased | (Delic et al., 2014) |  |
|  |  | LOPE | 12-14  18-20  26-28 | Serum | Biochemical analyzer | No significant change but differed in severe LOPE | (Villa et al., 2013) |  |
|  |  | PE at 34-37 weeks and >37 weeks | 20, 24, 28, 32, 36 | Serum | Biochemical analyzer | Significantly changed from 28 weeks in 34-37 weeks PE and 24 weeks in 37 weeks PE | (Leanos-Miranda et al., 2012) |  |
|  |  | Term PE | Longitudinal | Serum | ELISA | Increased level of sFlt-1 was associated with a higher risk for term PE | (Vatten et al., 2012) |  |
| Angiogenic and antiangiogenic protein | sFlt-1/PlGF ratio | LOPE | 11 - 20  Time of diagnosis | Serum | ELISA | Significantly increased  SHS: AUC = 0.88, 76.3% sensitivity at 83.2% specificity; OHS: AUC = 0.82, 70% sensitivity at 90.8% specificity | (Anto et al., 2021) |  |
|  |  | PE at 35-37 weeks and > 37 weeks | Longitudinal | Serum | Biochemical analyzer | Higher sFlt-1/PlGF levels (P < 0.001) in 35-37 weeks PE; this association was not seen in > 37 weeks PE. | (Fillion et al., 2020) |  |
|  |  | LOPE | 32-37 | Serum | Biochemical analyzer | AUC=0.93, 84.4% at 93% specificity | (Birdir et al., 2018) |  |
|  |  | LOPE | 9 - 15  20 - 42 | Serum | Biochemical analyzer | Increased at 9 -15 weeks | (Jaaskelainen et al., 2018) |  |
|  |  | LOPE | 20, 24, 28 | Serum | Biochemical analyzer | Significantly changed at 28 weeks | (Perales et al., 2017) |  |
|  |  | LOPE | 20-34 | Serum | Biochemical analyzer | AUC=0.639, 64% sensitivity at 63.7% specificity | (Andersen et al., 2016) |  |
|  |  | Term PE | Longitudinal | Serum | Biochemical analyzer | The ratio was significantly higher from 21 weeks onward. | (Khalil et al., 2016) |  |
|  |  | LOPE | 20-41 | Serum | Biochemical analyzer | Significantly increased | (Delic et al., 2014) |  |
|  |  | LOPE | 12-14  18-20  26-28 | Serum | Biochemical analyzer | No significant change in all LOPE cases, but differed in severe LOPE | (Villa et al., 2013) |  |
|  |  | PE at 34-37 weeks and >37 weeks | 20, 24, 28, 32, 36 | Serum | Biochemical analyzer | Significantly changed from 28 weeks in 34-37 weeks and >37 weeks PE | (Leanos-Miranda et al., 2012) |  |
| Angiogenic and antiangiogenic protein | Soluble emdoglin, sEng | LOPE | 11 - 20  Time of diagnosis | Serum | ELISA | Significantly increased  SHS: AUC = 0.75, 78.6% sensitivity at 84.2% specificity; OHS: AUC = 0.75, 63.3% sensitivity at 89.8% specificity | (Anto et al., 2021) |  |
|  |  | LOPE | 9 - 15  20 - 42 | Serum | ELISA | Increased at 9 -15 weeks | (Jaaskelainen et al., 2018) |  |
|  |  | Term PE | Longitudinal | Plasma | ELISA | sEng did not differ significantly from that of the normotensive group | (Khalil et al., 2014) |  |
| Angiogenic and antiangiogenic protein | Vascular endothelial growth factor A, VEGF-A | LOPE | 11 - 20  Time of diagnosis | Serum | ELISA | Significantly decreased  SHS: AUC=0.81, 69.1% sensitivity at 86.3% specificity; OHS: AUC=0.50, 73.3% sensitivity at 65.5% specificity | (Anto et al., 2021) |  |
| Angiogenic and antiangiogenic protein | Angiopoietin-2, Ang-2 | Term PE | Longitudinal | Plasma | ELISA | The level did not differ significantly from that in the control group | (Khalil et al., 2014) |  |
| Placenta-related protein | Pregnancy-associated plasma protein A, PAPP-A | LOPE | 32-37 | Serum | Biochemical analyzer | AUC=0.63, 50% at 80.2% specificity | (Birdir et al., 2018) |  |
|  |  | LOPE | 11 - 13 | Serum | ELISA | Significantly decreased | (Nuriyeva et al., 2017) |  |
|  |  | LOPE | 11- 13 | Serum | Not available | Significantly decreased with AUC = 0.751 | (Ceylan et al., 2014) |  |
|  |  | LOPE | 8-14 | Plasma | Biochemical analyzer | No significant difference | (Karahasanovic et al., 2014) |  |
|  |  | LOPE | 11-13 | serum | Biochemical analyzer | Significantly decreased | (Yu et al., 2013) |  |
| Placenta-related protein | Beta Human chorionic gonadotropin, β-HCG | LOPE | 8-14 | Plasma | Biochemical analyzer | Significant lower compared to healthy pregnancies | (Karahasanovic et al., 2014) |  |
| Placenta-related protein | Placental protein 13, PP-13 | LOPE | 11-13 | Serum | Biochemical analyzer | No significantly change | (Ceylan et al., 2014) |  |
| Placenta-related protein | A disintegrin and metalloprotease protein-12, ADAM12 | Term PE | 36 | Plasma | ELISA | Increased in the FLAG cohort with AUC=0.65, no significant changes in the BUMPS cohort; | (Andres et al., 2022) |  |
|  |  | LOPE | 11-13 | Serum | ELISA | No significant change in 11 - 13 weeks, DR is 18% at 10% FPR. Did not improve the prediction performance of combined model | (Kuc et al., 2013) |  |
| Placenta-related protein | Activin-A | Term PE | 28 and 36 | Plasma | ELISA | Increased at 36 weeks with AUC=0.71 | (Wong et al., 2022) |  |
|  |  | PE with delivery 34- 37 and ≥ 37 weeks | 11-13  30-33 | Serum | ELISA | No significant change at 11 – 13 weeks, but it increased at 30-33 weeks with AUC=0.722, DR=36% at 10% FPR | (Lai et al., 2013) |  |
| Placenta-related protein | Growth-differentiation factor 15, GDF-15 | Term PE | 36 | Plasma | ELISA | Significantly increased at 36 weeks in term PE with AUC=0.66 and 0.71 in two prospective cohorts | (Cruickshank et al., 2021) |  |
|  |  |  | 19-24  30-34  35-37 | Serum | ELISA | Significantly changed in preterm PE at 30-34 but no change in term PE | (Wertaschnigg et al., 2020) |  |
| Placenta-related protein | HtrA-1  HtrA3,  HtrA3-Long isoform | LOPE | 15 and 20 | Serum | ELISA | Decreased at 15 weeks with AUC=0.716, DR = 33.3% at 10% FPR; HtrA1 did not differ between the controls and cases. | (Teoh et al., 2019) |  |
|  |  |  | 11-13 | Serum |  | HtrA3-L increased at 11-13 weeks with AUC=0.61 | (Wang et al., 2018) |  |
| Placenta-related protein | Matrix metalloproteinase-7, MMP-7 | LOPE | Longitudinal | Plasma | Proteomics | MMP-7 is the best predictor of LOPE at 16-22 weeks with 70% DR at 20% FPR (AUC =0.82) | (Erez et al., 2017) |  |
|  |  |  | 11-14 | Serum | ELISA | Not statistically significant at 11-14 weeks with DR 14.8% at 10% FPR (AUC=0.548) | (Ravn et al., 2022) |  |
| Placenta-related protein | Inhibin-A | LOPE | 12-14  18-20  26-28 | Serum | ELISA | No significantly changed at 12-14 and 18-20, but it increased at 26-28 weeks with AUC=0.674 | (Keikkala et al., 2021) |  |
| Placenta-related protein | Pregnancy‐Specific β‐1 Glycoproteins 7 and 9, PSG7, PSG9 | Term PE | 36 | Plasma | ELISA | Both increased before the onset of term PE, PSG7 AUC=0.62, PSG9 AUC=0.65 | (Kandel et al., 2022) |  |
| Placenta-related protein | Tissue factor pathway inhibitor, TFPI | Term PE | 36 | Plasma | ELISA | Increased at 36 weeks prior to diagnosis of term PE with AUC=0.70, but not significantly changed at 28 weeks | (MacDonald et al., 2021) |  |
| Placenta-related protein | SPINT2 | Term PE | 36 | Plasma | ELISA | Increased at 36 weeks in term PE | (Murphy et al., 2021) |  |
| Placenta-related protein | Galectin-7 | LOPE | 10-12  17-20 | Serum | ELISA | Increased at 10-12 not at 17 – 20 weeks | (Menkhorst et al., 2014) |  |
| Other types of protein | Tumor necrosis factor receptor1, TNF-R1 | LOPE | 11-13  30-33 | Serum | Biochemical analyzer | Increased at 11-13 weeks, DR 20.9% at 10% FPR, AUC=0.531 | (SB et al., 2016) |  |
|  |  | LOPE | 11-13 |  | ELISA | TNF-R1 increased at 11-13 and 30-33 weeks. At 30-33 weeks, DR is 28% at 10% FPR, AUC=0.645 | (Mosimann et al., 2013) |  |
| Other types of protein | Cytokines | Term PE | 5-13  14-19 | Serum | Luminex MultiAnalyte Profiling | IL-1β is associated with term PE at 14-19 weeks | (Taylor, Ness, et al., 2016) |  |
|  |  |  | 9-26 weeks (mean 16 weeks) |  |  | IL-1β, IL-6 and TNF-β are significantly associated with term PE | (Taylor, Tang, et al., 2016) |  |
| Other types of protein | Macrophage migration inhibitory factor, MIF | LOPE | 5-18 | Serum | ELISA | No significantly difference | (Cardaropoli et al., 2014) |  |
| Other types of protein | Copeptin, CT-proAVP;  Mid-regional pro-atrial natriuretic peptide, MR-proANP;  Procalcitonin,PCT;  corin | LOPE | 32-37 | Serum | Biochemical analyzer | MR-proANP increased with AUC=0.72, 62.5% specificity at 87.1% specificity | (Birdir et al., 2020) |  |
|  |  | Term PE | Longitudinal | Plasma | ELISA | In term-PE groups, corin and MR-proANP levels did not differ significantly from the normotensive group | (Khalil et al., 2015) |  |
|  |  | LOPE | 11-13 | Serum | Biochemical analyzer | Copeptin (CT-proAVP), MR-proANP and PCT were not significantly different | (Birdir et al., 2015) |  |
|  |  | Term PE | Longitudinal  16, 22-32, 33-38 | Serum |  | Copeptin significantly increased eight weeks before diagnosis | (Yeung et al., 2014) |  |
| Other types of protein | Hydroxysteroid (17-β) dehydrogenase 1, HSD17B1 | LOPE | 20-23  27-30 | Plasma | ELISA | Decreased at 20-23 and 27-30 weeks | (Ishibashi et al., 2012) | |
| Other types of protein | ferritin light chain, FTL | LOPE | 11-13 | Plasma | ELISA | Decreased with AUC=0.685, 37.5% sensitivity at 97.62% specificity | (He et al., 2022) | |
| Other types of protein | ɑ1-microglobulin;  cell-free fetal hemoglobin, HbF | LOPE  Term PE | Mean 13.7 | Serum | ELISA, RIA | HbF increased in LOPE and term PE with AUC=0.66 and 0.68, DR are 23% and 19% at 10% FPR; A1M increased in LOPE and term PE with AUC=0.59 and 0.62, DR are 24% and 25% at 10 % FPR | (Anderson et al., 2016) | |
| Other types of protein | ELABELA | LOPE | 11-14 | Serum | ELISA | No significant difference | (Ozgen et al., 2021) | |
| Other types of protein | Endothelial Cell Specific Molecule 1, ESM-1 (Endocan) | LOPE | Longitudinal | Plasma | ELISA | ESM-1 was also decreased as  week 20 ± 2 until week 28 ± 2 of pregnancy. Then level increased and were no longer different on weeks 32 and 36 | (Schuitemaker et al., 2018) | |
| Other types of protein | Soluble suppression of tumorigenicity 2, soluble ST2 | LOPE | Longitudinal | Plasma | ELISA | Soluble ST2 increased after 33 weeks | (Romero et al., 2018) | |
| Other types of protein | Soluble LIGHT | LOPE | 18-24  27-31 | Serum | ELISA | sLIGHT at 27-31 weeks were independent risk factors for late-onset PE | (Hirashima et al., 2018) | |
| Other types of protein | Podocalyxin | LOPE | 11-13 | Serum | ELISA | Increased at 11-13 weeks with DR 11.11% at 10% FPR, AUC=0.692 | (Mansilla et al., 2018) | |
| Other types of protein | Afamin | LOPE | 11-13 | Serum | ELISA | Increased with 65.5% DR at 77.6% specificity, AUC=0.747 | (Koninger et al., 2018) | |
| Other types of protein | Leptin | Term PE | 9-26 | Serum | Luminex MultiAnalyte Profiling | Increased in term than in preterm PE | (Taylor et al., 2015) | |
| Other types of protein | Laeverin (aminopeptidase Q) | Term PE | 11-13 | Serum | ELISA | No significantly difference | (Pihl et al., 2019) | |
| Other types of protein | Retinol-binding protein 4, RBP4 | Term PE | Longitudinal | Plasma | ELISA | RBP4 was not associated with preeclampsia at term | (Mendola et al., 2017) | |
| Nucleic acid marker | Adrenomedullin (ADM) mRNA | Term PE | 28, 36 | Whole blood | RT-PCT | Case control: ADM mRNA decreased at 28 and 36 weeks with AUC = 0.64, and AUC=0.72 at 36 weeks  FLAG 1000 samples: AUC = 0.62 at 36 weeks | (Whigham et al., 2019) | |
| Nucleic acid marker | *PLAC 1* mRNA | LOPE | 14-18 | Plasma | RT-PCR | Increased with AUC=0.706, DR 30.2% | (Zanello et al., 2014) | |
| Nucleic acid marker | *HDC, MS4A2, SLC18A2* mRNA | LOPE | 10-12 | Whole blood | RT-PCR | Expressions of three genes are decreased in LOPE | (Lin et al., 2022) | |
| Nucleic acid marker | Methylated HYP2 level (m-HYP2) | LOPE | 15-19  24-28  33-41 | Plasma | RT-PCR | No significantly changed at second trimester but increased after onset | (Kwak et al., 2020) | |
| Nucleic acid marker | Fetal fraction (SeqFF) | LOPE | 11-13  20-24 | Plasma | Sequencing | No significantly changed at 11 – 13 but SeqFF decreased at 20-24 weeks | (Rolnik et al., 2015) | |
| Nucleic acid marker | mtDNA copy number | LOPE | 11-13 | Whole blood | RT-PCR | Decreased with AUC=0.82, 54% DR at 100% specificity | (Busnelli et al., 2019) | |
| Nucleic acid marker | miR-23b, miR-99b | LOPE | 11-13 | Plasma | RT-PCR | Down-regulated > fold change 1.5 with AUC=0.49 (miR-23b) and AUC=0.55 (miR-99) | (Mavreli et al., 2020) | |
| Nucleic acid marker | miR149, miR363 | Term PE | 28 and 36 | Whole blood | RT-PCR | miR18a, miR363, miR1283, miR149, miR16, miR424 reduced at 36 weeks; miR363 decreased both at 28 and 36 weeks | (Whigham et al., 2020) | |
| Nucleic acid marker | A panel of miRNAs | LOPE | 11-13 | PBMCs | RT-PCR | 30 miRNAs panel with AUC=0.90 and seven miRNAs panel with AUC=0.92 | (Winger et al., 2014, 2015) | |
| Metabolic marker | 8-epi-prostaglandin F2α, 8-epiPGF2α; 8-Hydroxy-2'-deoxyguanosine, 8-OHdG; total antioxidant capacity,  TAC | LOPE | 10-20 | Serum, Plasma, Urine | ELISA | Serum 8-epiPGF2α, urinary and serum 8-OHdG increased, and plasma TAC decreased at 10-20 weeks | (Anto et al., 2021) | |
| Metabolic marker | Uric acid | LOPE  Term PE | Longitudinal | Serum | Biochemical analyzer | Uric acid ratio increased after 20 weeks in those term PE with IUGR and raised in LOPE without IUGR after 30 weeks | (Corominas et al., 2021) | |
| Metabolic marker | Lathosterol, β­Sitosterol | LOPE | Longitudinal | Serum | LC-MS/MS | Lathosterol increased in LOPE compared to high-risk pregnancies at first trimester. β­Sitosterol and campersterol decreased in second trimester | (Antonic et al., 2021) | |
| Metabolic marker | Trace elements (K, Rb, and Ba) | Term PE | 36 | Plasma | ICP-MS | Changed at 36 weeks | (McKeating et al., 2021) | |
| Metabolic marker | 4-hydroxyglutamate, C-glycosyltryptophan | Term PE | 36 | Serum | Untargeted UPLC-MS | 100 metabolites differed at 20/28 wkGA and 33 metabolites validated at 36 weeks; 4-hydroxyglutamate and C-glycosyltryptophan were most predictive at 36 weeks | (Sovio et al., 2020) | |
| Metabolic marker | Isoleucine; decanoycarnitine; 1-Heptadecanoyl-2-hydroxy-sn-glycero-3 phosphocholine, 1-HGP | Term PE | 14 -16 | Plasma | LC-MS/MS | Isoleucine, decanoycarnitine and 1-HGP changed with AUC=0.61, 0.60, 0.61, at 7.4% FDR | (Kenny et al., 2020) | |
| Metabolic marker | Leukotriene B4, LTB4;  15-hydroxyeicosatetraenoic acid, 15(S)-HETE | Term PE | 19-24  30-34  35-37 | Serum | ELISA | LTB4 and 15(S)-HETE unchanged in term PE | (Santos et al., 2021) | |
| Metabolic marker | Vitamin D | LOPE | 11-13 | Serum | LC-MS/MS | No significantly change | (Yu et al., 2013) | |
| Metabolic macrker | Serum thiol/disulfide | LOPE | 11-13 | Serum | Automated spectrophotometric method | No significantly change | (Tasan et al., 2021) | |

PE, preeclampsia; GA, gestational age; LOPE, late-onset preeclampsia ≥ 34 weeks; term PE, term preeclampsia ≥ 37 weeks; ELISA, Enzyme-linked immunosorbent assay; AUC, Area under the ROC Curve; SGA, Small for gestational age; DR, detection rate; FPR, false positive rate; RIA, radioimmunoassay; RT-PCR, Reverse transcription polymerase chain reaction; PBMCs, peripheral blood mononuclear cells; LC-MS/MS, liquid chromatography (LC) tandem mass spectrometry (MS); UPLC-MS, Ultra-high performance liquid-chromatography-mass spectrometry

**Reference**

Andersen, L. B., Dechend, R., Jorgensen, J. S., Luef, B. M., Nielsen, J., Barington, T., & Christesen, H. T. (2016). Prediction of preeclampsia with angiogenic biomarkers. Results from the prospective Odense Child Cohort. *Hypertens Pregnancy*, *35*(3), 405-419. <https://doi.org/10.3109/10641955.2016.1167219>

Anderson, U. D., Gram, M., Ranstam, J., Thilaganathan, B., Kerstrom, B., & Hansson, S. R. (2016). Fetal hemoglobin, alpha1-microglobulin and hemopexin are potential predictive first trimester biomarkers for preeclampsia. *Pregnancy Hypertens*, *6*(2), 103-109. <https://doi.org/10.1016/j.preghy.2016.02.003>

Andres, F., Wong, G. P., Walker, S. P., MacDonald, T. M., Keenan, E., Cannon, P., Nguyen, T. V., Hannan, N. J., Tong, S., & Kaitu'u-Lino, T. J. (2022). A disintegrin and metalloproteinase 12 (ADAM12) is reduced at 36 weeks' gestation in pregnancies destined to deliver small for gestational age infants. *Placenta*, *117*, 1-4. <https://doi.org/10.1016/j.placenta.2021.11.001>

Anto, E. O., Coall, D. A., Addai-Mensah, O., Wiafe, Y. A., Owiredu, W., Obirikorang, C., Annani-Akollor, M. E., Adua, E., Tawiah, A., Acheampong, E., Asamoah, E. A., Wang, X., Opoku, S., Boakye, D. K., Hou, H., Wang, Y., Wang, W., & Suboptimal Health Study, C. (2021). Early gestational profiling of oxidative stress and angiogenic growth mediators as predictive, preventive and personalised (3P) medical approach to identify suboptimal health pregnant mothers likely to develop preeclampsia. *Epma j*, *12*(4), 517-534. <https://doi.org/10.1007/s13167-021-00258-x>

Antonic, T. D., Ardalic, D. C., Vladimirov, S. S., Banjac, G. S., Cabunac, P. J., Zeljkovic, A. R., Karadzov-Orlic, N. T., Spasojevic-Kalimanovska, V. V., Mikovic, Z. D., & Stefanovic, A. Z. (2021). Cholesterol homeostasis is dysregulated in women with preeclampsia. *Pol Arch Intern Med*, *131*(12). <https://doi.org/10.20452/pamw.16144>

Birdir, C., Droste, L., Fox, L., Frank, M., Fryze, J., Enekwe, A., Koninger, A., Kimmig, R., Schmidt, B., & Gellhaus, A. (2018). Predictive value of sFlt-1, PlGF, sFlt-1/PlGF ratio and PAPP-A for late-onset preeclampsia and IUGR between 32 and 37 weeks of pregnancy. *Pregnancy Hypertens*, *12*, 124-128. <https://doi.org/10.1016/j.preghy.2018.04.010>

Birdir, C., Fox, L., Droste, L., Frank, M., Fryze, J., Enekwe, A., Koninger, A., Kimmig, R., Schmidt, B., & Gellhaus, A. (2020). MR-proANP, a cardiovascular biomarker to predict late-onset preeclampsia and intrauterine growth restricted fetuses. *Pregnancy Hypertens*, *22*, 54-58. <https://doi.org/10.1016/j.preghy.2020.07.004>

Birdir, C., Janssen, K., Stanescu, A. D., Enekwe, A., Kasimir-Bauer, S., Gellhaus, A., Kimmig, R., & Köninger, A. (2015). Maternal serum copeptin, MR-proANP and procalcitonin levels at 11-13 weeks gestation in the prediction of preeclampsia. *Arch Gynecol Obstet*, *292*(5), 1033-1042. <https://doi.org/10.1007/s00404-015-3745-7>

Busnelli, A., Lattuada, D., Ferrari, S., Reschini, M., Colciaghi, B., Somigliana, E., Fedele, L., & Ferrazzi, E. (2019). Mitochondrial DNA Copy Number in Peripheral Blood in the First Trimester of Pregnancy and Different Preeclampsia Clinical Phenotypes Development: A Pilot Study. *Reprod Sci*, *26*(8), 1054-1061. <https://doi.org/10.1177/1933719118804410>

Cardaropoli, S., Ietta, F., Romagnoli, R., Rolfo, A., Paulesu, L., & Todros, T. (2014). Lower macrophage migration inhibitory factor concentrations in maternal serum before pre-eclampsia onset. *J Interferon Cytokine Res*, *34*(7), 537-542. <https://doi.org/10.1089/jir.2013.0057>

Ceylan, N., Ozaksit, G., Unlu, B. S., Yildiz, Y., Yilmaz, S., & Agaca, F. (2014). Can first trimester placental protein-13 and pregnancy-associated plasma protein-A predict pre-eclampsia in Turkish women? *J Obstet Gynaecol*, *34*(6), 482-485. <https://doi.org/10.3109/01443615.2014.911832>

Corominas, A. I., Medina, Y., Balconi, S., Casale, R., Farina, M., Martinez, N., & Damiano, A. E. (2021). Assessing the Role of Uric Acid as a Predictor of Preeclampsia. *Front Physiol*, *12*, 785219. <https://doi.org/10.3389/fphys.2021.785219>

Cruickshank, T., MacDonald, T. M., Walker, S. P., Keenan, E., Dane, K., Middleton, A., Kyritsis, V., Myers, J., Cluver, C., Hastie, R., Bergman, L., Garcha, D., Cannon, P., Murray, E., Nguyen, T. V., Hiscock, R., Pritchard, N., Hannan, N. J., Tong, S., & Kaitu'u-Lino, T. J. (2021). Circulating Growth Differentiation Factor 15 Is Increased Preceding Preeclampsia Diagnosis: Implications as a Disease Biomarker. *J Am Heart Assoc*, *10*(16), e020302. <https://doi.org/10.1161/JAHA.120.020302>

Delic, R., Stefanovic, M., Krivec, S., & Weber, V. (2014). Statistical regression model of standard and new laboratory markers and its usefulness in prediction of preeclampsia. *J Matern Fetal Neonatal Med*, *27*(4), 388-392. <https://doi.org/10.3109/14767058.2013.818121>

Erez, O., Romero, R., Maymon, E., Chaemsaithong, P., Done, B., Pacora, P., Panaitescu, B., Chaiworapongsa, T., Hassan, S. S., & Tarca, A. L. (2017). The prediction of late-onset preeclampsia: Results from a longitudinal proteomics study. *PLoS One*, *12*(7), e0181468. <https://doi.org/10.1371/journal.pone.0181468>

Fillion, A., Guerby, P., Lachance, C., Comeau, M. P., Bussieres, M. C., Doucet-Gingras, F. A., Zerounian, S., Demers, S., Laforest, G., Menzies, D., & Bujold, E. (2020). Placental Growth Factor and Soluble, Fms-Like Tyrosine Kinase-1 in Preeclampsia: A Case-Cohort (PEARL) Study. *J Obstet Gynaecol Can*, *42*(10), 1235-1242. <https://doi.org/10.1016/j.jogc.2020.03.024>

He, A., Wang, J., Yang, X., Liu, J., Yang, X., Wang, G., & Li, R. (2022). Screening of differentially expressed proteins in placentas from patients with late-onset preeclampsia. *Proteomics Clin Appl*, *16*(2), e2100053. <https://doi.org/10.1002/prca.202100053>

Hirashima, C., Ohmaru-Nakanishi, T., Nagayama, S., Takahashi, K., Suzuki, H., Takahashi, H., Usui, R., Shirasuna, K., Matsubara, S., & Ohkuchi, A. (2018). Serum soluble LIGHT in the early third trimester as a novel biomarker for predicting late-onset preeclampsia. *Pregnancy Hypertens*, *14*, 174-176. <https://doi.org/10.1016/j.preghy.2018.10.004>

Ishibashi, O., Ohkuchi, A., Ali, M. M., Kurashina, R., Luo, S. S., Ishikawa, T., Takizawa, T., Hirashima, C., Takahashi, K., Migita, M., Ishikawa, G., Yoneyama, K., Asakura, H., Izumi, A., Matsubara, S., Takeshita, T., & Takizawa, T. (2012). Hydroxysteroid (17-beta) dehydrogenase 1 is dysregulated by miR-210 and miR-518c that are aberrantly expressed in preeclamptic placentas: a novel marker for predicting preeclampsia. *Hypertension*, *59*(2), 265-273. <https://doi.org/10.1161/HYPERTENSIONAHA.111.180232>

Jaaskelainen, T., Heinonen, S., Hamalainen, E., Pulkki, K., Romppanen, J., Laivuori, H., & Finnpec. (2018). Angiogenic profile in the Finnish Genetics of Pre-Eclampsia Consortium (FINNPEC) cohort. *Pregnancy Hypertens*, *14*, 252-259. <https://doi.org/10.1016/j.preghy.2018.03.004>

Kandel, M., MacDonald, T. M., Walker, S. P., Cluver, C., Bergman, L., Myers, J., Hastie, R., Keenan, E., Hannan, N. J., Cannon, P., Nguyen, T. V., Pritchard, N., Tong, S., & Kaitu'u-Lino, T. J. (2022). PSG7 and 9 (Pregnancy-Specific beta-1 Glycoproteins 7 and 9): Novel Biomarkers for Preeclampsia. *J Am Heart Assoc*, *11*(7), e024536. <https://doi.org/10.1161/JAHA.121.024536>

Karahasanovic, A., Sorensen, S., & Nilas, L. (2014). First trimester pregnancy-associated plasma protein A and human chorionic gonadotropin-beta in early and late pre-eclampsia. *Clin Chem Lab Med*, *52*(4), 521-525. <https://doi.org/10.1515/cclm-2013-0338>

Keikkala, E., Forsten, J., Ritvos, O., Stenman, U. H., Kajantie, E., Hamalainen, E., Raikkonen, K., Villa, P. M., & Laivuori, H. (2021). Serum Inhibin-A and PAPP-A2 in the prediction of pre-eclampsia during the first and second trimesters in high-risk women. *Pregnancy Hypertens*, *25*, 116-122. <https://doi.org/10.1016/j.preghy.2021.05.024>

Kenny, L. C., Thomas, G., Poston, L., Myers, J. E., Simpson, N. A. B., McCarthy, F. P., Brown, L. W., Bond, A. E., Tuytten, R., Baker, P. N., & Screening for Pregnancy Endpoints, C. (2020). Prediction of preeclampsia risk in first time pregnant women: Metabolite biomarkers for a clinical test. *PLoS One*, *15*(12), e0244369. <https://doi.org/10.1371/journal.pone.0244369>

Khalil, A., Maiz, N., Garcia-Mandujano, R., Elkhouli, M., & Nicolaides, K. H. (2014). Longitudinal changes in maternal soluble endoglin and angiopoietin-2 in women at risk for pre-eclampsia. *Ultrasound Obstet Gynecol*, *44*(4), 402-410. <https://doi.org/10.1002/uog.13439>

Khalil, A., Maiz, N., Garcia-Mandujano, R., Elkhouli, M., & Nicolaides, K. H. (2015). Longitudinal changes in maternal corin and mid-regional proatrial natriuretic peptide in women at risk of pre-eclampsia. *Ultrasound Obstet Gynecol*, *45*(2), 190-198. <https://doi.org/10.1002/uog.14685>

Khalil, A., Maiz, N., Garcia-Mandujano, R., Penco, J. M., & Nicolaides, K. H. (2016). Longitudinal changes in maternal serum placental growth factor and soluble fms-like tyrosine kinase-1 in women at increased risk of pre-eclampsia. *Ultrasound Obstet Gynecol*, *47*(3), 324-331. <https://doi.org/10.1002/uog.15750>

Koninger, A., Enekwe, A., Mach, P., Andrikos, D., Schmidt, B., Frank, M., Birdir, C., Kimmig, R., Gellhaus, A., & Dieplinger, H. (2018). Afamin: an early predictor of preeclampsia. *Arch Gynecol Obstet*, *298*(5), 1009-1016. <https://doi.org/10.1007/s00404-018-4897-z>

Kuc, S., Koster, M. P., Franx, A., Schielen, P. C., & Visser, G. H. (2013). Maternal characteristics, mean arterial pressure and serum markers in early prediction of preeclampsia. *PLoS One*, *8*(5), e63546. <https://doi.org/10.1371/journal.pone.0063546>

Kwak, D. W., Kim, S. Y., Kim, H. J., Lim, J. H., Kim, Y. H., & Ryu, H. M. (2020). Maternal total cell-free DNA in preeclampsia with and without intrauterine growth restriction. *Sci Rep*, *10*(1), 11848. <https://doi.org/10.1038/s41598-020-68842-1>

Lai, J., Pinas, A., Syngelaki, A., Poon, L. C., & Nicolaides, K. H. (2013). Maternal serum activin-A at 30-33 weeks in the prediction of preeclampsia. *J Matern Fetal Neonatal Med*, *26*(8), 733-737. <https://doi.org/10.3109/14767058.2012.755167>

Leanos-Miranda, A., Campos-Galicia, I., Isordia-Salas, I., Rivera-Leanos, R., Romero-Arauz, J. F., Ayala-Mendez, J. A., & Ulloa-Aguirre, A. (2012). Changes in circulating concentrations of soluble fms-like tyrosine kinase-1 and placental growth factor measured by automated electrochemiluminescence immunoassays methods are predictors of preeclampsia. *J Hypertens*, *30*(11), 2173-2181. <https://doi.org/10.1097/HJH.0b013e328357c0c9>

Lin, J., Meng, Y., Song, M. F., & Gu, W. (2022). Network-Based Analysis Reveals Novel Biomarkers in Peripheral Blood of Patients With Preeclampsia. *Front Mol Biosci*, *9*, 757203. <https://doi.org/10.3389/fmolb.2022.757203>

MacDonald, T. M., Tong, S., Myers, J., Cannon, P., Nguyen, T. V., Keenan, E., Murray, E., Harper, A., Pritchard, N., Hannan, N. J., Dane, K. M., Middleton, A. L., Kyritsis, V. P., Walker, S. P., & Kaitu'u-Lino, T. J. (2021). Circulating Tissue Factor Pathway Inhibitor (TFPI) is increased preceding preeclampsia diagnosis and in established preeclampsia. *Placenta*, *105*, 32-40. <https://doi.org/10.1016/j.placenta.2021.01.018>

Mansilla, M., Wang, Y., Hyett, J., da Silva Costa, F., & Nie, G. (2018). Serum podocalyxin for early detection of preeclampsia at 11-13 weeks of gestation. *Placenta*, *71*, 13-15. <https://doi.org/10.1016/j.placenta.2018.09.009>

Mavreli, D., Lykoudi, A., Lambrou, G., Papaioannou, G., Vrachnis, N., Kalantaridou, S., Papantoniou, N., & Kolialexi, A. (2020). Deep Sequencing Identified Dysregulated Circulating MicroRNAs in Late Onset Preeclampsia. *In Vivo*, *34*(5), 2317-2324. <https://doi.org/10.21873/invivo.12044>

McKeating, D. R., Fisher, J. J., MacDonald, T., Walker, S., Tong, S., Bennett, W. W., Kaitu'u-Lino, T. J., & Perkins, A. V. (2021). Circulating trace elements for the prediction of preeclampsia and small for gestational age babies. *Metabolomics*, *17*(10), 90. <https://doi.org/10.1007/s11306-021-01840-0>

Mendola, P., Ghassabian, A., Mills, J. L., Zhang, C., Tsai, M. Y., Liu, A., & Yeung, E. H. (2017). Retinol-Binding Protein 4 and Lipids Prospectively Measured During Early to Mid-Pregnancy in Relation to Preeclampsia and Preterm Birth Risk. *Am J Hypertens*, *30*(6), 569-576. <https://doi.org/10.1093/ajh/hpx020>

Menkhorst, E., Koga, K., Van Sinderen, M., & Dimitriadis, E. (2014). Galectin-7 serum levels are altered prior to the onset of pre-eclampsia. *Placenta*, *35*(4), 281-285. <https://doi.org/10.1016/j.placenta.2014.01.009>

Mosimann, B., Wagner, M., Birdir, C., Poon, L. C., & Nicolaides, K. H. (2013). Maternal serum tumour necrosis factor receptor 1 (TNF-R1) at 30-33 weeks in the prediction of preeclampsia. *J Matern Fetal Neonatal Med*, *26*(8), 763-767. <https://doi.org/10.3109/14767058.2012.755168>

Murphy, C. N., Walker, S. P., MacDonald, T. M., Keenan, E., Hannan, N. J., Wlodek, M. E., Myers, J., Briffa, J. F., Romano, T., Roddy Mitchell, A., Whigham, C. A., Cannon, P., Nguyen, T. V., Kandel, M., Pritchard, N., Tong, S., & Kaitu'u-Lino, T. J. (2021). Elevated Circulating and Placental SPINT2 Is Associated with Placental Dysfunction. *Int J Mol Sci*, *22*(14). <https://doi.org/10.3390/ijms22147467>

Nucci, M., Poon, L. C., Demirdjian, G., Darbouret, B., & Nicolaides, K. H. (2014). Maternal serum placental growth factor isoforms 1 and 2 at 11-13, 20-24 and 30-34 weeks' gestation in late-onset pre-eclampsia and small for gestational age neonates. *Fetal Diagn Ther*, *35*(4), 249-257. <https://doi.org/10.1159/000358595>

Nuriyeva, G., Kose, S., Tuna, G., Kant, M., Akis, M., Altunyurt, S., Islekel, G. H., & Dogan, O. E. (2017). A prospective study on first trimester prediction of ischemic placental diseases. *Prenat Diagn*, *37*(4), 341-349. <https://doi.org/10.1002/pd.5017>

Ozgen, G., Aydin, G. A., & Ayvaci, H. (2021). Predictive Role of First-trimester ELABELA Levels for Late-onset Preeclampsia. *J Coll Physicians Surg Pak*, *31*(8), 916-920. <https://doi.org/10.29271/jcpsp.2021.08.916>

Palmer, K. R., Kaitu'u-Lino, T. J., Cannon, P., Tuohey, L., De Silva, M. S., Varas-Godoy, M., Acuña, S., Galaz, J., Tong, S., & Illanes, S. E. (2017). Maternal plasma concentrations of the placental specific sFLT-1 variant, sFLT-1 e15a, in fetal growth restriction and preeclampsia. *J Matern Fetal Neonatal Med*, *30*(6), 635-639. <https://doi.org/10.1080/14767058.2016.1182975>

Perales, A., Delgado, J. L., de la Calle, M., Garcia-Hernandez, J. A., Escudero, A. I., Campillos, J. M., Sarabia, M. D., Laiz, B., Duque, M., Navarro, M., Calmarza, P., Hund, M., Alvarez, F. V., & investigators, S. (2017). sFlt-1/PlGF for prediction of early-onset pre-eclampsia: STEPS (Study of Early Pre-eclampsia in Spain). *Ultrasound Obstet Gynecol*, *50*(3), 373-382. <https://doi.org/10.1002/uog.17373>

Pihl, K., Sorensen, S., Nystad, M., Acharya, G., & Jorgensen, F. S. (2019). Maternal serum laeverin (aminopeptidase Q) measured in the first trimester of pregnancy does not predict preeclampsia. *J Matern Fetal Neonatal Med*, *32*(20), 3348-3351. <https://doi.org/10.1080/14767058.2018.1463368>

Ravn, J. D., Bendix, E. J., Sperling, L., & Overgaard, M. (2022). First trimester serum matrix metalloproteinase-7 is a poor predictor of late-onset preeclampsia. *Pregnancy Hypertens*, *28*, 94-99. <https://doi.org/10.1016/j.preghy.2022.03.002>

Rolnik, D. L., O'Gorman, N., Fiolna, M., van den Boom, D., Nicolaides, K. H., & Poon, L. C. (2015). Maternal plasma cell-free DNA in the prediction of pre-eclampsia. *Ultrasound Obstet Gynecol*, *45*(1), 106-111. <https://doi.org/10.1002/uog.14671>

Romero Infante, X. C., Uriel, M., Rincon Franco, S., Ibanez Pinilla, E. A., & Rojas, N. A. (2022). First trimester placental growth factor in maternal blood and placenta related disorders. *J Matern Fetal Neonatal Med*, *35*(25), 7668-7675. <https://doi.org/10.1080/14767058.2021.1960966>

Romero, R., Chaemsaithong, P., Tarca, A. L., Korzeniewski, S. J., Maymon, E., Pacora, P., Panaitescu, B., Chaiyasit, N., Dong, Z., Erez, O., Hassan, S. S., & Chaiworapongsa, T. (2018). Maternal plasma-soluble ST2 concentrations are elevated prior to the development of early and late onset preeclampsia - a longitudinal study. *J Matern Fetal Neonatal Med*, *31*(4), 418-432. <https://doi.org/10.1080/14767058.2017.1286319>

Santos, L. L., Wertaschnigg, D., Rolnik, D. L., Da Silva Costa, F., Syngelaki, A., Dimitriadis, E., & Nicolaides, K. H. (2021). Serum leukotriene B4 and hydroxyeicosatetraenoic acid in the prediction of pre-eclampsia. *Placenta*, *103*, 76-81. <https://doi.org/10.1016/j.placenta.2020.10.007>

SB, E. H. M., Park, F., Murthi, P., Martins, W. P., Kane, S. C., Williams, P., Hyett, J., & da Silva Costa, F. (2016). TNF-R1 as a first trimester marker for prediction of pre-eclampsia. *J Matern Fetal Neonatal Med*, *29*(6), 897-903. <https://doi.org/10.3109/14767058.2015.1022865>

Schuitemaker, J. H. N., Cremers, T., Van Pampus, M. G., Scherjon, S. A., & Faas, M. M. (2018). Changes in endothelial cell specific molecule 1 plasma levels during preeclamptic pregnancies compared to healthy pregnancies. *Pregnancy Hypertens*, *12*, 58-64. <https://doi.org/10.1016/j.preghy.2018.02.012>

Sovio, U., McBride, N., Wood, A. M., Masconi, K. L., Cook, E., Gaccioli, F., Charnock-Jones, D. S., Lawlor, D. A., & Smith, G. C. S. (2020). 4-Hydroxyglutamate is a novel predictor of pre-eclampsia. *Int J Epidemiol*, *49*(1), 301-311. <https://doi.org/10.1093/ije/dyz098>

Tasan, H. A., Aydin, G. A., Cakar, E., Usal, N. T., Cogendez, E., Ozkaya, E., Bicer, C., & Erel, O. (2021). The Predictive Value of First-trimester Thiol/Disulfide Homeostasis in Preeclampsia. *J Coll Physicians Surg Pak*, *30*(4), 405-409. <https://doi.org/10.29271/jcpsp.2021.04.405>

Taylor, B. D., Ness, R. B., Klebanoff, M. A., Zoh, R., Bass, D., Hougaard, D. M., Skogstrand, K., & Haggerty, C. L. (2016). First and second trimester immune biomarkers in preeclamptic and normotensive women. *Pregnancy Hypertens*, *6*(4), 388-393. <https://doi.org/10.1016/j.preghy.2016.09.002>

Taylor, B. D., Ness, R. B., Olsen, J., Hougaard, D. M., Skogstrand, K., Roberts, J. M., & Haggerty, C. L. (2015). Serum leptin measured in early pregnancy is higher in women with preeclampsia compared with normotensive pregnant women. *Hypertension*, *65*(3), 594-599. <https://doi.org/10.1161/HYPERTENSIONAHA.114.03979>

Taylor, B. D., Tang, G., Ness, R. B., Olsen, J., Hougaard, D. M., Skogstrand, K., Roberts, J. M., & Haggerty, C. L. (2016). Mid-pregnancy circulating immune biomarkers in women with preeclampsia and normotensive controls. *Pregnancy Hypertens*, *6*(1), 72-78. <https://doi.org/10.1016/j.preghy.2015.11.002>

Teoh, S. S. Y., Wang, Y., Li, Y., Leemaqz, S. Y., Dekker, G. A., Roberts, C. T., & Nie, G. (2019). Low Serum Levels of HtrA3 at 15 Weeks of Gestation Are Associated with Late-Onset Preeclampsia Development and Small for Gestational Age Birth. *Fetal Diagn Ther*, *46*(6), 392-401. <https://doi.org/10.1159/000497144>

Vatten, L. J., Asvold, B. O., & Eskild, A. (2012). Angiogenic factors in maternal circulation and preeclampsia with or without fetal growth restriction. *Acta Obstet Gynecol Scand*, *91*(12), 1388-1394. <https://doi.org/10.1111/j.1600-0412.2012.01516.x>

Villa, P. M., Hamalainen, E., Maki, A., Raikkonen, K., Pesonen, A. K., Taipale, P., Kajantie, E., & Laivuori, H. (2013). Vasoactive agents for the prediction of early- and late-onset preeclampsia in a high-risk cohort. *Bmc Pregnancy and Childbirth*, *13*. <https://doi.org/Artn> 110

10.1186/1471-2393-13-110

Wang, Y., Li, Y., Hyett, J., da Silva Costa, F., & Nie, G. (2018). HtrA3 Isoform-Specific ELISAs for Early Detection of Preeclampsia. *SLAS Discov*, *23*(10), 1092-1099. <https://doi.org/10.1177/1087057116682425>

Wertaschnigg, D., Rolnik, D. L., Nie, G., Teoh, S. S. Y., Syngelaki, A., da Silva Costa, F., & Nicolaides, K. H. (2020). Second- and third-trimester serum levels of growth-differentiation factor-15 in prediction of pre-eclampsia. *Ultrasound Obstet Gynecol*, *56*(6), 879-884. <https://doi.org/10.1002/uog.22070>

Whigham, C. A., MacDonald, T. M., Walker, S. P., Hiscock, R., Hannan, N. J., Pritchard, N., Cannon, P., Nguyen, T. V., Miranda, M., Tong, S., & Kaitu'u-Lino, T. J. (2020). MicroRNAs 363 and 149 are differentially expressed in the maternal circulation preceding a diagnosis of preeclampsia. *Sci Rep*, *10*(1), 18077. <https://doi.org/10.1038/s41598-020-73783-w>

Whigham, C. A., MacDonald, T. M., Walker, S. P., Pritchard, N., Hannan, N. J., Hastie, R., Alwis, N., Cannon, P., Nguyen, T. V., Tong, S., & Kaitu'u-Lino, T. (2019). Circulating adrenomedullin mRNA is decreased in women destined to develop term preeclampsia. *Pregnancy Hypertens*, *16*, 16-25. <https://doi.org/10.1016/j.preghy.2019.02.003>

Winger, E. E., Reed, J. L., & Ji, X. (2014). First trimester PBMC microRNA predicts adverse pregnancy outcome. *Am J Reprod Immunol*, *72*(5), 515-526. <https://doi.org/10.1111/aji.12287>

Winger, E. E., Reed, J. L., & Ji, X. (2015). First-trimester maternal cell microRNA is a superior pregnancy marker to immunological testing for predicting adverse pregnancy outcome. *J Reprod Immunol*, *110*, 22-35. <https://doi.org/10.1016/j.jri.2015.03.005>

Wong, G. P., Andres, F., Walker, S. P., MacDonald, T. M., Cannon, P., Nguyen, T. V., Keenan, E., Hannan, N. J., Tong, S., & Kaitu'u-Lino, T. J. (2022). Circulating Activin A is elevated at 36 weeks' gestation preceding a diagnosis of preeclampsia. *Pregnancy Hypertens*, *27*, 23-26. <https://doi.org/10.1016/j.preghy.2021.11.006>

Yeung, E. H., Liu, A., Mills, J. L., Zhang, C., Männistö, T., Lu, Z., Tsai, M. Y., & Mendola, P. (2014). Increased levels of copeptin before clinical diagnosis of preeclampsia. *Hypertension*, *64*(6), 1362-1367. <https://doi.org/10.1161/hypertensionaha.114.03762>

Yu, C. K. H., Ertl, R., Skyfta, E., Akolekar, R., & Nicolaides, K. H. (2013). Maternal serum vitamin D levels at 11-13 weeks of gestation in preeclampsia. *Journal of Human Hypertension*, *27*(2), 115-118. <https://doi.org/10.1038/jhh.2012.1>

Zanello, M., Sekizawa, A., Purwosunu, Y., Curti, A., & Farina, A. (2014). Circulating mRNA for the PLAC1 gene as a second trimester marker (14-18 weeks' gestation) in the screening for late preeclampsia. *Fetal Diagn Ther*, *36*(3), 196-201. <https://doi.org/10.1159/000360854>
