# Supplementary material for: Potential biomarkers for late-onset and term preeclampsia: A scoping review
Source: Front Physiol. 2023 Mar 10;14:1143543. doi: 10.3389/fphys.2023.1143543 (PMC10036383; doi:10.3389/fphys.2023.1143543)
Supplement: Supplementary file 2 [file Table2.DOCX]

**Table s2. Characteristics of prediction models combined maternal factors with biochemical and/or biophysical markers in late and term PE**

| **Study** | **Country** | **PE subtypes** | **Population** | **Study Design** | **Sample**  **size** | **Biochemical marker** | **Screen GA**  **(weeks)** | **Combined model** | **Algorithm** | **DR at 10%FPR** | **AUC** | **Note** |
| --- | --- | --- | --- | --- | --- | --- | --- | --- | --- | --- | --- | --- |
| (Chaiyasit et al., 2022) | Hongkong | LOPE with delivery ≥ 34 weeks | Multicenter cohort study of singleton pregnancies recruited at 11-13 weeks’ at six maternity units in Asia, Dec 2016 - Dec 2018: China, Hong Kong SAR, Thailand, and Singapore | Prospective cohort | 7877 | PlGF  sFlt-1 | 11-13 | MF+SBP+PlGF+sFlt-1 | IPCC reported model | 28.2% | 0.669 | Competing risks model for the prediction of any-onset and late-onset PE perform  better than the IPCC reported model |
|  |  |  |  |  |  |  |  | MF+MAP+PlGF | Competing risks model | 48.1% | 0.789 |  |
| (Gana et al., 2022) | UK | Term PE with delivery ≥ 37 weeks | From a routine hospital visit in the UK between Jun and Aug 2019, May 2020, and Feb 2021 | Prospective cohort | 4066 | PAPP-A  PlGF | 11-13 | MF + MAP + UtA-PI + PlGF + PSV ratio | Competing risks model | 47% | NA | PAPP-A is not superior to PlGF; PSV ratio did not improve the prediction of term PE (46.7% DR without PSV ratio) |
| (Ravn et al., 2022) | Denmark | LOPE with delivery ≥ 34 weeks | From a routine first-trimester scan at Odense University Hospital, 13 Aug 2019 - Nov 2019 | Case-control | 27 LOPEs  194 controls | MMP7 ^ns^ | 11-14 | MF + MMP7 | Logistic regression | 51.9% | 0.831 | MMP-7 alone is not a useful predictor (DR 14.8%). Adding MMP-7 to any combination of variables did not improve the detection rate |
|  |  |  |  |  |  |  |  | MAP + MMP-7 |  | 44.4% | 0.815 |  |
| (Keikkala et al., 2021) | Finland | LOPE diagnosis ≥ 34 weeks | From Prediction and Prevention of Pre-eclampsia and Intrauterine Growth Restriction (PREDO) project high-risk population, 2005 - 2009 | Case-control | 11 EOPEs  34 LOPEs 89 controls | 12- 14 and 18- 20 weeks: Inhibin-A^ns^ PAPP-A2^ns^  PAPP-A^ns^  PlGF^ns^  26-28 weeks: Inhibin-A^a^ PAPP-A2^a^  PAPP-A^ns^  PlGF^a^ | 12 -14 | BMI + prior PE + Inhibin-A + UtA-PI | Logistic regression | NA | 0.824 | Inhibin-A shows the potential ability to predict EOPE and LOPE |
| (Pihl et al., 2020) | Denmark | Term PE with delivery ≥ 37 weeks | From a cohort of first-trimester combined screening program for trisomy 21 from 1 Jan 2005 to 31 Dec 2010 at the Copenhagen University Hospital | Case-control | 55 preterm PEs  213 term PEs  449 controls | PAPP-A^ns^,  β-hCG^ns^, PlGF^a^,  sFlt-1^ns^,  NT-proBNP^a^, NGAL^ns^ | 11-13 | MF+MAP+BMI+PlGF | Logistic regression | 32.7% | 0.666 | These predictors, individually or in combination, are less useful for term PE |
| (Post Uiterweer et al., 2020) | Netherlands,USA | LOPE with delivery ≥ 34 weeks | Pittsburgh group: a pilot study at University of Pittsburgh, Pittsburgh, PA, USA; Dutch group: large Dutch nested case-control group collected in Netherlands | Case-control | Pittsburgh group: 33 LOPEs, 25 controls; Dutch group: 57 EOPEs, 95 LOPEs, 469 controls | Relaxin | 11-13 | MF + MAP + Relaxin | Logistic regression | 45.1% | 0.801 | Relaxin is not a clinically useful biomarker for LOPE |
| (Mazer Zumaeta et al., 2020) | UK | Term PE with delivery ≥ 37 weeks | From prospective screening for adverse obstetric outcomes in women with singleton pregnancy attending a routine first-trimester hospital visit in the UK, 2006 - 2017 | Prospective cohort | 60875 | PAPP-A  PlGF | 11-13 | MF + MAP + UtA-PI + PlGF | Competing- risks model | 44% | NA | The addition of serum PAPP-A does not improve the prediction of PE provided by maternal factors, MAP, UtA-PI and PlGF |
| (Boutin et al., 2019) | Canada | Term PE with delivery ≥ 37 weeks | From nulliparous women with singleton pregnancy at the time of ultrasound in Quebec City, Canada, Mar 2011 - Dec 2014 | Prospective cohort | 4652 | PAPP-A PlGF | 11-13 | MF+ PlGF | Proportional hazard models | 26% | 0.66 | DR of PlGF alone is 21% (AUC = 0.61%). Moreover, PAPP-A did not significantly improve the prediction of term PE |
| (Zhang et al., 2019) | China | LOPE with delivery ≥ 34 weeks | From first-trimester trisomy screening in China, Dec 2016 - May 2018 | Prospective cohort | 3270 | PlGF^a^, PAPP-A^a^ | 11-13 | MF + MAP + PlGF + PAPP-A | Commercial software from Perkin Elmer | 48.57% | 0.828 | LOPE with SGA achieved a higher DR than without SGA |
| (Orosz et al., 2019) | Hungary | LOPE with delivery ≥ 34 weeks | From a prospective cohort of low-risk pregnancies in Hungary | Case-control | 11 EOPE, 71 LOPE, 82 controls | PAPP-A  PlGF | 11-13 | MF + MAP + UtA-PI + PAPP-A | Commercial software from Astraia Software GmBH | Astraia 2.3.2: 64.8% | NA | PlGF did not improve the efficacy |
|  |  |  |  |  |  |  |  |  |  | Astraia 2.8.1: 54.9% |  |  |
| (Tan, Wright, et al., 2018) | UK | Term PE with delivery ≥ 37 weeks | A prospective multicentre cohort study, carried out in seven maternity hospitals in the UK, 12 Apr - 15 Dec 2016 | Prospective cohort | 16747 | PAPP-A  PlGF | 11-13 | MF + MAP + UtA-PI + PAPP-A + PlGF | Competing- risks model | 43.5% | NA | Combined MF with biomarkers is superior to NICE guidelines |
| (Tan, Syngelaki, et al., 2018) | UK | Term PE with delivery ≥ 37 weeks | The data were derived from three previously reported prospective non-intervention screening studies in a combined total of 61 174 singleton pregnancies | Re-analysis of cohort studies | 61174 | PAPP-A  PlGF | 11-13 | MF + MAP + UtA-PI + PlGF | Competing-risks model | 41.0% | 0.776 | PAPP-A did not improve the performance of combined screening (DR 41.3%) |
| (Sonek et al., 2018) | USA | LOPE  with delivery ≥ 34 weeks; term PE ≥ 37 weeks | From unselected US Patients who presented for an ultrasound examination from 2013 to 2016 | Prospective cohort | 1288 | AFP^ns^  PAPP-A^ns^  PlGF^ns^ | 11-13 | MF+PAPP-A+AFP+PlGF+MAP+UtA-PI+EPV | Logistic regression | LOPE: 36% | NA | Biomarkers did not improve the DR for LOPE and term PE |
|  |  |  |  |  |  |  |  |  |  | Term PE: 29% |  |  |
| (Murtoniemi et al., 2018) | Finland | LOPE diagnosed ≥ 34 weeks | A subcohort of high-risk women in the prospectively collected PREDO cohort, Sep 2005 - Jun 2009 | Case-control | 9 EOPEs,  25 LOPEs, 223 controls | %hCG-h^a^  β-hCG^ns^  hCG-h^ns^  PAPP-A^ns^ PlGF^ns^ | 11-13 | MF+MAP+UtA-PI+ hCG+%hCG-h, β-hCG + PlGF | Logistic regression | 32% | 0.66 | %hCG-h levels significantly reduced in LOPE but less useful in the prediction model |
| (Guizani et al., 2018) | Belgium | Term PE with delivery ≥ 37 weeks | Singleton pregnancies undergoing first-trimester screening for Down's syndrome, Jan 2011- Dec 2013 | Prospective cohort | 3239 | PAPP-A PlGF | 11-13 | MF+MAP+UtPI+PAPP-A+PlGF | Competing risks model | 31.8% | 0.741 | FMF algorithm validation |
| (Cheng et al., 2018) | China | LOPE with delivery ≥ 34 weeks | From the first trimester Down syndrome screening test in Hongkong, Aug 2013 - Apr 2014 | Case-control | 3330 | PlGF | 11-13 | MF+MAP+UtA-PI+PlGF | Logistic regression | 55% | 0.72 | Compared to FMF model |
| (R. Bahado-Singh et al., 2017) | UK | Term PE with delivery ≥ 37 weeks | First and third trimester screening of the general obstetric population for prediction of obstetric and fetal complications | Case-control | 35 term PEs,  63 controls | *Metabolites* | 11-14 | Putrescine, Urea, Carnitine | Logistic regression | Sensitivity 72.7% at specificity 57.4% | 0.701 | First-only, third-only and the serial combination of metabolite and peptides significantly predicted term PE. |
|  |  |  |  |  |  |  | 30-34 | Methylhistidine, Serotonin, Citrate, Hexose, Propylene glycol |  | Sensitivity 74.2% at specificity 72.3% | 0.761 |  |
|  |  |  |  |  |  |  | 11-14 and 30-34 | Urea (1st), Hexose (3rd), SM C18:1 (1st), Citrate (3rd), MAP (32 wks), BMI (12 wks) |  | Sensitivity 84.2% at specificity 71.0% | 0.734 |  |
| (R. O. Bahado-Singh et al., 2017) | UK | LOPE with delivery ≥ 34 weeks | Part of prospective study being conducted by the Fetal Medicine Foundation London, England, for the first trimester prediction of pregnancy complications. | Case-control | 29 LOPEs, 55 controls | *Metabolites* | 11-13 | Weight + UtA-PI + pyruvate + carnitine | Logistic regression | 34.8% at specificity 82.6 | 0.734 | Predictive performance for late-PE detection is modest. |
| (Scazzocchio et al., 2017) | Spain | LOPE with delivery ≥ 34 weeks | A validation cohort of 4621 consecutive women attending their routine first-trimester ultrasound examination was used to test a prediction model for PE that had been developed previously in 5170 women. | Validation cohort | 4203 | PAPP-A,  β-hCG | 11-13 | MF+MAP+UtA-PI+PAPP-A | Logistic regression | 43.3% | 0.720 | Validation of a previous construction cohort (2013) |
| (O'Gorman et al., 2017) | UK, Spain, Belgium, Greece, Italy | Term PE with delivery ≥ 37 weeks | From 12 maternity hospitals in five different countries, Feb - Sep 2015 | Prospective cohort | 8775 | PlGF  PAPP-A | 11-13 | MF+MAP+UtA-PI+PlGF | Competing risks model | 43% | 0.792 | FMF algorithm slightly improved the detection rate compared to maternal factors only; the addition of PAPP-A did not improve term PE |
| (O'Gorman et al., 2016) | UK | Term PE with delivery ≥ 37 weeks | Prospective screening for adverse obstetric outcomes in women attending routine hospital visits in the UK, Feb 2010 - Jul 2014 | Prospective cohort | 35948 | PAPP-A  PlGF | 11-13 | MF+UtA-PI+MAP+PlGF | Competing risks model | 47% | NA | PAPP-A did not improve the performance of the screening |
| (SB et al., 2016) | Brazil | LOPE with delivery ≥ 34 weeks | Unselected population attending an inner-city tertiary obstetric hospital for antenatal care, Apr 2011 - Mar 2012 | Case-control | 6 EOPEs  43 LOPEs  66 GHs  311 controls | TNF-R1^ns^ | 11-13 | MF+TNF-R1 | Logistic regression | 18.6% | 0.701 | TNFR-1 did not improve the detection rate of LOPE compared with maternal factors alone |
| (Crovetto, Figueras, Triunfo, et al., 2015) | Spain | LOPE with delivery ≥ 34 weeks | From a large prospective cohort of unselected singleton pregnancies referring for routine first-trimester screening for aneuploidies | Case-control | 57 EOPEs,  246 LOPEs,  9159 controls | PAPP-A^a^,  β-hCG^ns^,  PlGF^a^,  sFlt-1^a^,  sFlt-1/PlGF ratio^a^ | 11-13 | MF+MAP+UtA-PI+PlGF+sFlt-1 | Logistic regression | 76.4% | 0.871 | Angiogenic factors improved the prediction |
| (Crovetto, Figueras, Crispi, et al., 2015) | Spain | LOPE with delivery ≥ 34 weeks | From a large prospective cohort of 5,759 unselected singleton pregnancies referred for routine first-trimester screening for aneuploidies at the Department of Maternal-Fetal Medicine at the Hospital Clinic Barcelona, Spain | Case-control | 20 EOPEs, 20 LOPEs,  300 controls | β-HCG^ns^, PAPP-A^ns^  PlGF^a^  sFlt-1^ns^  sFlt-1/PlGF ratio^a^  hCG-sLHCGR^ns^ LHCGR-D/R^ns^ | 8-13 | MF+UtA-PI+PlGF+sFlt-1+hCG-LHCGR+sLHCGR | Logistic regression | 75% | 0.923 | LHCGR forms improved the prediction for LOPE (DR increased by 6–15%) |
| (Koster et al., 2015) | Netherland | LOPE with delivery ≥ 34 weeks | From a large cohort of women participating in the routine Dutch first-trimester prenatal screening, 2007 - 2009 | Case-control | 68 EOPE, 99 LOPE,  500 controls | PAPP  PlGF  *acylcarnitines^a^* | 11-13 | Prior risk + MAP + PAPP-A + PlGF + stearoylcarnitine | Logistic regression | Training set: 58% | Training set: 0.833 | Stearoyl carnitine is a novel metabolomic marker for LOPE |
|  |  |  |  |  |  |  |  |  |  | Validation: 32% | Validation: 32% |  |
| (Skrastad et al., 2015) | Norway | LOPE with delivery ≥ 34 weeks | Participants attended a study visit between 11-13 weeks at the National Centre for Fetal Medicine, St Olavs Hospital, Trondheim, Norway, Sep 2010 -Mar 2012 | Prospective cohort | 585 | PAPP-A  PlGF | 11-13 | MF+MAP+UtA-PI+PlGF+PAPP-A | Competing risks model | 30% | 0.739 | Two algorithms had similar prediction performance |
|  |  |  |  |  |  |  |  |  | Commercial software from Perkin Elmer | 30% | 0.713 |  |
| (Crovetto et al., 2014) | Spain | LOPE with delivery ≥ 34 weeks | From a large prospective cohort of 5,759 unselected singleton pregnancies referring for routine first-trimester screening for aneuploidies at the Department of Maternal-Fetal Medicine at Hospital Clinic Barcelona | Case-control | 28 EOPEs, 84 LOPEs, 84 controls | PAPP-A^ns^  β-hCG^ns^ PlGF^a^  sFlt-1^a^  sFlt-1/PlGF ratio^a^  sEng^a^ | 11-13 | MF+UtA-PI+PlGF+sFlt-1 | Logistic regression | 69% | 0.888 | sFlt-1 significantly increased in first trimester and sEng did not improve the combined model |
| (Kuc et al., 2014) | Netherlands | LOPE with delivery ≥ 34 weeks | From a large cohort of women participating in the routine Dutch first trimester Down syndrome screening, 2007 - 2009 | Case-control | 68 EOPEs, 99 LOPEs, 500 controls | *Metabolites* | 8-13 | Prior risk + MAP + glycylglycine | Logistic regression | Training set: 53% | Training set: 0.830 | Although glycylglycine significantly decreased in LOPE, it did not improve the prediction model |
|  |  |  |  |  |  |  |  |  |  | Test set: 42% | Test set: 0.780 |  |
| (Teixeira et al., 2014) | Portugal | LOPE depending on when findings first become apparent ≥ 34 weeks | From routine first trimester aneuploidy screening in a Portuguese university hospital, Jan 2009 - Jun 2013 | Retrospective cohort | 5672 | PAPP-A^a^,  β-hCG^ns^ | 9-13 | MF+aneuploidy screening biomarkers (CRL, NT, PAPP-A, β-hCG) | Logistic regression | 35.2% | 0.734 | Aneuploidy screening is not useful for LOPE |
| (Bahado-Singh et al., 2013) | UK | LOPE with delivery ≥ 34 weeks | Part of an ongoing prospective study being conducted by the Fetal Medicine Foundation, London, UK, for the first-trimester prediction of important fetal and obstetric disorders, 2003 - 2009 | Case-control | 30 LOPEs, 59 controls | *Metabolites* | 11-13 | Valine, weight, race, and others (pyruvate, Hydroxybutyrate_3, 1-methylhistidine, glycerol, trimethylamine) | Logistic regression | 76.6% at specificity 100% | 0.960 | Significant differences in the first-trimester metabolites were noted in LOPE and between EOPE and LOPE |
| (Kuc et al., 2013) | Netherlands | LOPE with delivery ≥ 34 weeks | From a large cohort of women participating in the routine Dutch first-trimester Down syndrome screening, 2007 - 2009 | Case-control | 68 EOPEs, 99 LOPEs, 500 controls | PAPP-A^ns^, hCGβ^ns^, PlGF^ns^, ADAM12^ns^ | 11-13 | MF+MAP+PAPP-A+PlGF+ADAM12 | Logistic regression | 49% | NA | DR of ADAM12 alone is 18% and it did not improve the combined screening. |
| (Park et al., 2013) | Australia | LOPE with delivery ≥ 34 weeks | An unselected cohort who opted for first trimester screening in Australia, Apr 2010 - Mar 2012 | Retrospective cohort | 3099 | PAPP-A^a^ | 11-13 | MF+MAP+PAPP-A | Logistic regression | 32.4 % | 0.760 | Maternal factor with MAP and UtA-PI performed better than with PAPP-A (DR 35.2%) |
| (Parra-Cordero et al., 2013) | Chile | LOPE with delivery ≥ 34 weeks | From 5367 asymptomatic pregnant women who underwent a routine scan in 1st trimester, Apr 2002 - Jul 2010 | Case-control | 17 EOPE,  53 LOPE, 289 controls | PlGF^a^,  sFlt-1^ns^, sEng^ns^ | 11-13 | MF+UtA-PI+PlGF | Logistic regression | 29.4% | NA |  |
| (Scazzocchio et al., 2013) | Spain | LOPE with delivery ≥ 34 weeks | A prospective cohort composed of singleton pregnancies underwent routine first-trimester screening at the Department of Maternal-Fetal Medicine at Hospital Clinic Barcelona, May - Oct 2011 | Prospective cohort | 5759 | PAPP-A^a^, HCG^ns^ | 8-13 | MF+MAP+UtA-PI+PAPP-A | Logistic regression | 39.6% | 0.710 |  |
| (Di Lorenzo et al., 2012) | Italy | LOPE diagnosed ≥ 34 weeks | Recruited from Oct 2007 to Apr 2009 and were followed from first trimester ultrasound aneuploidy screening | Prospective cohort | 2118 | β-HCG^ns^, PAPP-A^ns^, PlGF^ns^,  PP-13^ns^ | 11-13 | MF+UtA-PI+HCG+PAPP-A+PlGF | Model A: FMF regression analysis; | Both are 31% | NA |  |
|  |  |  |  |  |  |  |  |  | Model B: logistic regression |  |  |  |
| (Abdelaziz et al., 2012) | Saudi Arabia | LOPE with delivery ≥ 34 weeks | Women attending routine antenatal care in the Kingdom of Saudi Arabia, Oct 2009 - Dec 2010 | Case-control | 16 EOPEs,  13 LOPEs,  13 GHs,  178 controls | sEng | 11-14 | MAP+UtA-PI+sEng | Logistic regression | 80.3% | 0.83 | DR of sEng alone is 28.3%, AUC=0.59 |
| (Sapantzoglou et al., 2021) | UK | Term with delivery ≥ 37 weeks | From a routine hospital visit at King's College Hospital, London, UK, Aug 2019 - Apr 2020 | Prospective cohort | 2853 | PlGF  sFlt-1 | 19-23 | MF + MAP + UtA-PI + PlGF | Competing- risks model | 43.0% | 0.810 | PSV ratio modestly improved the prediction of term PE; biochemical markers are less useful |
|  |  |  |  |  |  |  |  | MF + MAP + UtA-PI + PlGF + PSV ratio |  | 53.4% | 0.859 |  |
| (Teoh et al., 2019) | Australia | LOPE occurring at ≥ 34 weeks | Recruited at the Lyell McEwin hospital to the Adelaide cohort of the SCOPE study | Case-control | 92 PEs  71 GHs  56 Preterms 116 SGAs  330 controls | HtrA1^ns^  HtrA3^a^  PAPP-A | 15-20 | HtrA3 + Uterine D + umb D+ PAPP-A (12 wks) | Logistic regression | 38.6% | 0.755 | HtrA3 may be useful in predicting LOPE at 15 weeks |
| (Gallo et al., 2016) | UK | Term PE with delivery ≥ 37 weeks | From prospective screening for adverse obstetric outcomes in women attending routine pregnancy care in the UK, Jan 2006 - Jul 2014 | Prospective cohort | 7748 | PlGF  sFlt-1 | 19-24 | MF+MAP+UtA-PI+PlGF | Competing- risks model | 46% | 0.801 | The DR was not improved by the addition of sFlt-1 |
| (Zanello et al., 2014) | Italy | LOPE occurring after 34 weeks | The women were examined between mid-2005 and 2006 and comprised singleton pregnancies that visited the Department of Obstetrics and Gynaecology, University of Indonesia, at Cipto Mangunkusumo National Hospital. | Case-control | 43 LOPEs 200 controls | *PLAC1 mRNA* | 14-18 | MF+MAP+PLAC (mRNA) | Logistic regression | 62.8% | 0.866 | Screening combining maternal factors with PLAC1 is useful for predicting late PE. PLAC1 alone yielded a DR of 30.2% |
| (Lai, Pinas, Poon, et al., 2013) | UK | Intermediate PE with delivery at 34 - 37 weeks term PE with delivery ≥ 37 weeks | Prospective observational study for adverse pregnancy outcomes in women attending for their routine first- and third-trimester hospital visits in pregnancy in the UK, May 2011 - Mar 2012 | Case-control | 50 LOPEs  250 controls | 11-13 weeks  PlGF^a^ PAPP-A^a^  β-hCG^ns^  30-33 weeks PlGF^a^ PAPP-A^ns^  β-hCG^a^ | 30-33 | MF+PlGF | Logistic regression | Intermediate PE; 85.7%  term PE  52.8% | Intermediate PE: 0.939; term PE  0.783 | Screening by MF and serum PlGF at 30–33 weeks could identify the most late PE cases. Addition of free β-hCG did not improve the performance of screening |
| (Lai, Pinas, Syngelaki, et al., 2013) | UK | LOPE with delivery ≥ 34 weeks | Prospective observational study for adverse pregnancy outcomes in women attending for their routine first- and third-trimester hospital visits in pregnancy in the UK, May 2011 - Mar 2012 | Case-control | 50 LOPEs  250 controls | 11-13 weeks Activin-A^ns^  30-33 weeks  Activin-A^a^ | 30-33 | MF+Activin-A | Logistic regression | 50% | 0.772 | Combining maternal characteristics and activin-A at 30–33 weeks could identify half of LOPE |
| (Lai, Syngelaki, et al., 2013) | UK | Intermediate PE with delivery at 34 – 37 weeks; term PE with delivery ≥ 37 weeks | Prospective observational study for adverse pregnancy outcomes in women attending for their routine first- and third-trimester hospital visits in pregnancy in the UK, May 2011 - Mar 2012 | Case-control | 50 LOPEs  250 controls | 11-13 weeks sEng^ns^  30-33 weeks  sEng ^a^ | 30-33 | MF+sEng | Logistic regression | Intermediate PE: 64.3%;  term PE:  50% | Intermediate PE: 0.908; term PE:  0.762 | sEng at 30–33 weeks combined with MF could identify half of LOPE |
| (Mosimann et al., 2013) | UK | LOPE with delivery ≥ 34 weeks | Prospective observational study for adverse pregnancy outcomes in women attending for their routine first- and third-trimester hospital visits in pregnancy in the UK, May 2011 - Mar 2012 | Case-control | 50 LOPEs  250 controls | 11-13 weeks TNF-R1^a^  30-33 weeks  TNF-R1^a^ | 30-33 | MF+TNF-R1 | Logistic regression | 40% | 0.777 | TNF-R1 was increased at both 11–13 and 30–33 weeks but may be only useful at 30 - 33 |
| (Tsiakkas et al., 2016) | UK | Term PE with delivery ≥ 37 weeks | Prospective screening for adverse obstetric outcomes in women attending their routine hospital visit in the third trimester of pregnancy in UK, Mar 2011 - Dec 2014 | Prospective cohort | 7927 | PlGF  sFlt-1 | 30-34 | MF+MAP+UtA-PI+PlGF+sFlt-1 | Competing- risks model | 66% | 0.875 | A combination of maternal factors and biomarkers in the early third trimester could predict over half of those with term PE, which is superior to that achieved by screening in the first and second trimesters with respective DRs of about 41% and 46% for term-PE |
| (Valino et al., 2016a) | UK | Term PE with delivery ≥ 37 weeks | Prospective screening for adverse obstetric outcomes in women attending their routine hospital visit in the third trimester of pregnancy in the UK, May 2011 - Aug 2014 | Prospective cohort | 8268 | PlGF^a^  sFlt-1^a^ | 30-34 | MCA-PI + MAP + PlGF + sFlt-1 | Logistic regression | 55.8% | 0.808 | The combined screening is useful for PE and SGA rather than other adverse outcomes such as stillbirth |
| (Dobert et al., 2022) | Multicentre in Europe | Term PE with delivery ≥ 37 weeks | STATIN study. a prospective, multicenter study in singleton pregnancies at late gestation for routine pregnancy care at 10 maternity hospitals | Prospective cohort | 29677 | PlGF  sFlt-1 | 35-37 | MF+MAP+ PlGF+sFlt-1 | Competing-risks model | 79% | 0.933 | The addition of UtA-PI did not improve the prediction (DR is 78%) |
| (Sarno et al., 2021) | UK | Term PE with delivery ≥ 37 weeks | From a routine visit at late gestation at King's College Hospital, London, UK, Jun 2019 - Mar 2020 | Prospective cohort | 2287 | PlGF  sFlt-1 | 35-37 | MF+MAP+ UtA-PI+PlGF+sFlt-1+PSV ratio | Competing- risks model | Delivery at any time: 77.2% | 0.901 | DR is higher in imminent PE with delivery within 3 weeks after the assessment. |
|  |  |  |  |  |  |  |  |  |  | Delivery within 3 weeks: 94% | 0.954 |  |
| (Ciobanu et al., 2019) | UK | Term PE with delivery ≥ 37 weeks | From a routine hospital visit at late gestation in the UK, Oct 2016 - Sep 2018 | Prospective cohort | 15,247 | PlGF  sFlt-1 | 35-37 | MF+MAP+PlGF+sFlt-1 | Competing- risks model | Delivery within 2 weeks  93% at 10% SPR | 0.975 | The triple test is superior to PlGF alone or sFlt-1/PlGF ratio |
|  |  |  |  |  |  |  |  |  |  | Delivery within 4 weeks  DR 72% at 10% SPR | 0.907 |  |
| (Panaitescu et al., 2018) | UK | Term PE with delivery ≥ 37 weeks | A prospective observational study in women attending a routine hospital visit at late gestation in the UK | Prospective cohort | 13350 | PlGF,  sFlt-1 | 35-37 | MF+MAP+PlGF+sFlt-1 | Competing- risks model | 70.2% at 9.7% FRP | NA | Screening by maternal factors and biomarkers at 35–37 weeks' gestation can identify a high proportion of term PE; UtA-PI did not improve the performance of screening |
| (Valino et al., 2016b) | UK | Term PE with delivery ≥ 37 weeks | Prospective screening for adverse obstetric outcomes in women attending their routine hospital visit in the third trimester of pregnancy in the UK, Feb 2014 - Dec 2014 | Prospective cohort | 3953 | PlGF^a^  sFlt-1^a^ | 35-37 | MAP+ PlGF+sFlt-1 | Logistic regression | 73.3% at | 0.913 | Prediction of most cases of term PE and SGA is best achieved by assessment at around 36 weeks |
| (Andrietti et al., 2016) | UK | Term PE with delivery ≥ 37 weeks | Prospective screening for adverse obstetric outcomes in women attending routine pregnancy care at 35 + 0to 37 + 6 weeks gestation in two maternity hospitals in the UK, Feb - Dec 2014 | Prospective cohort | 5362 | PlGF  sFlt-1 | 35-37 | MF+MAP+UtA-PI+PlGF+sFlt-1 | Competing-risks model | 84% | 0.939 | A combination of maternal factors and biomarkers at 35–37 weeks' gestation can provide effective screening for term PE. |

^a^ indicates the biomarker significantly changed; ^ns^ indicates the biomarker did not show significantly change.

PE, preeclampsia; GA, gestational age; DR, detection rate; FPR, false positive rate; AUC, area under the ROC curve; LOPE, late-onset preeclampsia; SBP, systolic blood pressure; PlGF, placental growth factor; sFlt-1, soluble fms-like tyrosine kinase-1; IPCC, International Prediction of Pregnancy Complications Network; MF, maternal factor; MAP, mean arterial pressure; UtA-PI, uterine artery pulsatility index; PSV ratio, peak systolic velocity ratio; PAPP-A, pregnancy-associated plasma protein A; MMP-7, matrix metalloproteinase-7; BMI, body mass index; NA, not available; EOPE, early-onset preeclampsia; β-hCG, beta human chorionic gonadotropin; NT-proBNP, N-terminal (NT)-pro Natriuretic Peptide; NGAL, neutrophil gelatinase-associated lipocalin; SGA, Small for gestational age; AFP, alpha-fetoprotein; FMF, fetal medicine foundation; TNF-R1, tumor necrosis factor receptor1; LHCGR, luteinizing hormone/choriogonadotropin receptor; CRL, crown rump length; NT, nuchal translucency; ADAM12, a disintegrin and metalloprotease protein-12; sEng, soluble endoglin; EPV, Estimated Placental Volume

**Reference**

Abdelaziz, A., Maher, M. A., Sayyed, T. M., Bazeed, M. F., & Mohamed, N. S. (2012). Early pregnancy screening for hypertensive disorders in women without a-priori high risk. *Ultrasound Obstet Gynecol*, *40*(4), 398-405. <https://doi.org/10.1002/uog.11205>

Andrietti, S., Silva, M., Wright, A., Wright, D., & Nicolaides, K. H. (2016). Competing-risks model in screening for pre-eclampsia by maternal factors and biomarkers at 35-37 weeks' gestation. *Ultrasound Obstet Gynecol*, *48*(1), 72-79. <https://doi.org/10.1002/uog.15812>

Bahado-Singh, R., Poon, L. C., Yilmaz, A., Syngelaki, A., Turkoglu, O., Kumar, P., Kirma, J., Allos, M., Accurti, V., Li, J., Zhao, P., Graham, S. F., Cool, D. R., & Nicolaides, K. (2017). Integrated Proteomic and Metabolomic prediction of Term Preeclampsia. *Sci Rep*, *7*(1), 16189. <https://doi.org/10.1038/s41598-017-15882-9>

Bahado-Singh, R. O., Akolekar, R., Mandal, R., Dong, E., Xia, J., Kruger, M., Wishart, D. S., & Nicolaides, K. (2013). First-trimester metabolomic detection of late-onset preeclampsia. *Am J Obstet Gynecol*, *208*(1), 58 e51-57. <https://doi.org/10.1016/j.ajog.2012.11.003>

Bahado-Singh, R. O., Syngelaki, A., Mandal, R., Graham, S. F., Akolekar, R., Han, B., Bjondahl, T. C., Dong, E., Bauer, S., Alpay-Savasan, Z., Turkoglu, O., Ogunyemi, D., Poon, L. C., Wishart, D. S., & Nicolaides, K. H. (2017). Metabolomic determination of pathogenesis of late-onset preeclampsia. *J Matern Fetal Neonatal Med*, *30*(6), 658-664. <https://doi.org/10.1080/14767058.2016.1185411>

Boutin, A., Demers, S., Gasse, C., Giguere, Y., Tetu, A., Laforest, G., & Bujold, E. (2019). First-Trimester Placental Growth Factor for the Prediction of Preeclampsia in Nulliparous Women: The Great Obstetrical Syndromes Cohort Study. *Fetal Diagn Ther*, *45*(2), 69-75. <https://doi.org/10.1159/000487301>

Chaiyasit, N., Sahota, D. S., Ma, R., Choolani, M., Wataganara, T., Sim, W. S., Chaemsaithong, P., Wah, Y. M. I., Hui, S. Y. A., & Poon, L. C. (2022). Prospective Evaluation of International Prediction of Pregnancy Complications Collaborative Network Models for Prediction of Preeclampsia: Role of Serum sFlt-1 at 11-13 Weeks' Gestation. *Hypertension*, *79*(2), 314-322. <https://doi.org/10.1161/HYPERTENSIONAHA.121.18021>

Cheng, Y., Leung, T. Y., Law, L. W., Ting, Y. H., Law, K. M., & Sahota, D. S. (2018). First trimester screening for pre-eclampsia in Chinese pregnancies: case-control study. *Bjog*, *125*(4), 442-449. <https://doi.org/10.1111/1471-0528.14970>

Ciobanu, A., Wright, A., Panaitescu, A., Syngelaki, A., Wright, D., & Nicolaides, K. H. (2019). Prediction of imminent preeclampsia at 35-37 weeks gestation. *Am J Obstet Gynecol*, *220*(6), 584 e581-584 e511. <https://doi.org/10.1016/j.ajog.2019.01.235>

Crovetto, F., Figueras, F., Crispi, F., Triunfo, S., Pugia, M., Lasalvia, L., Chambers, A. E., Mills, W. E., Banerjee, S., Mercade, I., Casals, E., Mira, A., Rodriguez-Revenga Bodi, L., & Gratacos, E. (2015). Forms of Circulating Luteinizing Hormone Human Chorionic Gonadotropin Receptor for the Prediction of Early and Late Preeclampsia in the First Trimester of Pregnancy. *Fetal Diagn Ther*, *38*(2), 94-102. <https://doi.org/10.1159/000371516>

Crovetto, F., Figueras, F., Triunfo, S., Crispi, F., Rodriguez-Sureda, V., Dominguez, C., Llurba, E., & Gratacos, E. (2015). First trimester screening for early and late preeclampsia based on maternal characteristics, biophysical parameters, and angiogenic factors. *Prenat Diagn*, *35*(2), 183-191. <https://doi.org/10.1002/pd.4519>

Crovetto, F., Figueras, F., Triunfo, S., Crispi, F., Rodriguez-Sureda, V., Peguero, A., Dominguez, C., & Gratacos, E. (2014). Added value of angiogenic factors for the prediction of early and late preeclampsia in the first trimester of pregnancy. *Fetal Diagn Ther*, *35*(4), 258-266. <https://doi.org/10.1159/000358302>

Di Lorenzo, G., Ceccarello, M., Cecotti, V., Ronfani, L., Monasta, L., Vecchi Brumatti, L., Montico, M., & D'Ottavio, G. (2012). First trimester maternal serum PIGF, free beta-hCG, PAPP-A, PP-13, uterine artery Doppler and maternal history for the prediction of preeclampsia. *Placenta*, *33*(6), 495-501. <https://doi.org/10.1016/j.placenta.2012.03.003>

Dobert, M., Wright, A., Varouxaki, A. N., Mu, A. C., Syngelaki, A., Rehal, A., Delgado, J. L., Akolekar, R., Muscettola, G., Janga, D., Singh, M., Martin-Alonso, R., Dutemeyer, V., De Alvarado, M., Atanasova, V., Wright, D., & Nicolaides, K. H. (2022). STATIN trial: predictive performance of competing-risks model in screening for pre-eclampsia at 35-37 weeks' gestation. *Ultrasound Obstet Gynecol*, *59*(1), 69-75. <https://doi.org/10.1002/uog.24789>

Gallo, D. M., Wright, D., Casanova, C., Campanero, M., & Nicolaides, K. H. (2016). Competing risks model in screening for preeclampsia by maternal factors and biomarkers at 19-24 weeks' gestation. *Am J Obstet Gynecol*, *214*(5), 619 e611-619 e617. <https://doi.org/10.1016/j.ajog.2015.11.016>

Gana, N., Sarno, M., Vieira, N., Wright, A., Charakida, M., & Nicolaides, K. H. (2022). Ophthalmic artery Doppler at 11-13 weeks' gestation in prediction of pre-eclampsia. *Ultrasound Obstet Gynecol*, *59*(6), 731-736. <https://doi.org/10.1002/uog.24914>

Guizani, M., Valsamis, J., Dutemeyer, V., Kang, X., Ceccotti, V., Khalife, J., Duiella, S. F., Blavier, F., Faraca, A., Cos, T., & Jani, J. C. (2018). First-Trimester Combined Multimarker Prospective Study for the Detection of Pregnancies at a High Risk of Developing Preeclampsia Using the Fetal Medicine Foundation-Algorithm. *Fetal Diagn Ther*, *43*(4), 266-273. <https://doi.org/10.1159/000477934>

Keikkala, E., Forsten, J., Ritvos, O., Stenman, U. H., Kajantie, E., Hamalainen, E., Raikkonen, K., Villa, P. M., & Laivuori, H. (2021). Serum Inhibin-A and PAPP-A2 in the prediction of pre-eclampsia during the first and second trimesters in high-risk women. *Pregnancy Hypertens*, *25*, 116-122. <https://doi.org/10.1016/j.preghy.2021.05.024>

Koster, M. P., Vreeken, R. J., Harms, A. C., Dane, A. D., Kuc, S., Schielen, P. C., Hankemeier, T., Berger, R., Visser, G. H., & Pennings, J. L. (2015). First-Trimester Serum Acylcarnitine Levels to Predict Preeclampsia: A Metabolomics Approach. *Dis Markers*, *2015*, 857108. <https://doi.org/10.1155/2015/857108>

Kuc, S., Koster, M. P., Franx, A., Schielen, P. C., & Visser, G. H. (2013). Maternal characteristics, mean arterial pressure and serum markers in early prediction of preeclampsia. *PLoS One*, *8*(5), e63546. <https://doi.org/10.1371/journal.pone.0063546>

Kuc, S., Koster, M. P., Pennings, J. L., Hankemeier, T., Berger, R., Harms, A. C., Dane, A. D., Schielen, P. C., Visser, G. H., & Vreeken, R. J. (2014). Metabolomics profiling for identification of novel potential markers in early prediction of preeclampsia. *PLoS One*, *9*(5), e98540. <https://doi.org/10.1371/journal.pone.0098540>

Lai, J., Pinas, A., Poon, L. C., Agathokleous, M., & Nicolaides, K. H. (2013). Maternal serum placental growth factor, pregnancy-associated plasma protein-a and free beta-human chorionic gonadotrophin at 30-33 weeks in the prediction of pre-eclampsia. *Fetal Diagn Ther*, *33*(3), 164-172. <https://doi.org/10.1159/000345090>

Lai, J., Pinas, A., Syngelaki, A., Poon, L. C., & Nicolaides, K. H. (2013). Maternal serum activin-A at 30-33 weeks in the prediction of preeclampsia. *J Matern Fetal Neonatal Med*, *26*(8), 733-737. <https://doi.org/10.3109/14767058.2012.755167>

Lai, J., Syngelaki, A., Poon, L. C., Nucci, M., & Nicolaides, K. H. (2013). Maternal serum soluble endoglin at 30-33 weeks in the prediction of preeclampsia. *Fetal Diagn Ther*, *33*(3), 149-155. <https://doi.org/10.1159/000343220>

Mazer Zumaeta, A., Wright, A., Syngelaki, A., Maritsa, V. A., Da Silva, A. B., & Nicolaides, K. H. (2020). Screening for pre-eclampsia at 11-13 weeks' gestation: use of pregnancy-associated plasma protein-A, placental growth factor or both. *Ultrasound Obstet Gynecol*, *56*(3), 400-407. <https://doi.org/10.1002/uog.22093>

Mosimann, B., Wagner, M., Birdir, C., Poon, L. C., & Nicolaides, K. H. (2013). Maternal serum tumour necrosis factor receptor 1 (TNF-R1) at 30-33 weeks in the prediction of preeclampsia. *J Matern Fetal Neonatal Med*, *26*(8), 763-767. <https://doi.org/10.3109/14767058.2012.755168>

Murtoniemi, K., Villa, P. M., Matomaki, J., Keikkala, E., Vuorela, P., Hamalainen, E., Kajantie, E., Pesonen, A. K., Raikkonen, K., Taipale, P., Stenman, U. H., & Laivuori, H. (2018). Prediction of pre-eclampsia and its subtypes in high-risk cohort: hyperglycosylated human chorionic gonadotropin in multivariate models. *BMC Pregnancy Childbirth*, *18*(1), 279. <https://doi.org/10.1186/s12884-018-1908-9>

O'Gorman, N., Wright, D., Poon, L. C., Rolnik, D. L., Syngelaki, A., Wright, A., Akolekar, R., Cicero, S., Janga, D., Jani, J., Molina, F. S., de Paco Matallana, C., Papantoniou, N., Persico, N., Plasencia, W., Singh, M., & Nicolaides, K. H. (2017). Accuracy of competing-risks model in screening for pre-eclampsia by maternal factors and biomarkers at 11–13 weeks' gestation. *Ultrasound in Obstetrics and Gynecology*, *49*(6), 751-755. <https://doi.org/10.1002/uog.17399>

O'Gorman, N., Wright, D., Syngelaki, A., Akolekar, R., Wright, A., Poon, L. C., & Nicolaides, K. H. (2016). Competing risks model in screening for preeclampsia by maternal factors and biomarkers at 11-13 weeks gestation. *AMERICAN JOURNAL OF OBSTETRICS AND GYNECOLOGY*, *214*(1). <https://doi.org/ARTN> 103.e1-e12

10.1016/j.ajog.2015.08.034

Orosz, L., Orosz, G., Veress, L., Dosa, D., Orosz, L., Sr., Arany, I., Fabian, A., Medve, L., Pap, K., Karanyi, Z., Toth, Z., Poka, R., Than, N. G., & Torok, O. (2019). Screening for preeclampsia in the first trimester of pregnancy in routine clinical practice in Hungary. *J Biotechnol*, *300*, 11-19. <https://doi.org/10.1016/j.jbiotec.2019.04.017>

Panaitescu, A., Ciobanu, A., Syngelaki, A., Wright, A., Wright, D., & Nicolaides, K. H. (2018). Screening for pre-eclampsia at 35-37 weeks' gestation. *Ultrasound Obstet Gynecol*, *52*(4), 501-506. <https://doi.org/10.1002/uog.19111>

Park, F. J., Leung, C. H., Poon, L. C., Williams, P. F., Rothwell, S. J., & Hyett, J. A. (2013). Clinical evaluation of a first trimester algorithm predicting the risk of hypertensive disease of pregnancy. *Aust N Z J Obstet Gynaecol*, *53*(6), 532-539. <https://doi.org/10.1111/ajo.12126>

Parra-Cordero, M., Rodrigo, R., Barja, P., Bosco, C., Rencoret, G., Sepulveda-Martinez, A., & Quezada, S. (2013). Prediction of early and late pre-eclampsia from maternal characteristics, uterine artery Doppler and markers of vasculogenesis during first trimester of pregnancy. *Ultrasound Obstet Gynecol*, *41*(5), 538-544. <https://doi.org/10.1002/uog.12264>

Pihl, K., Sorensen, S., & Stener Jorgensen, F. (2020). Prediction of Preeclampsia in Nulliparous Women according to First Trimester Maternal Factors and Serum Markers. *Fetal Diagn Ther*, *47*(4), 277-283. <https://doi.org/10.1159/000503229>

Post Uiterweer, E. D., Koster, M. P. H., Jeyabalan, A., Kuc, S., Siljee, J. E., Stewart, D. R., Conrad, K. P., & Franx, A. (2020). Circulating pregnancy hormone relaxin as a first trimester biomarker for preeclampsia. *Pregnancy Hypertens*, *22*, 47-53. <https://doi.org/10.1016/j.preghy.2020.07.008>

Ravn, J. D., Bendix, E. J., Sperling, L., & Overgaard, M. (2022). First trimester serum matrix metalloproteinase-7 is a poor predictor of late-onset preeclampsia. *Pregnancy Hypertens*, *28*, 94-99. <https://doi.org/10.1016/j.preghy.2022.03.002>

Sapantzoglou, I., Wright, A., Arozena, M. G., Campos, R. V., Charakida, M., & Nicolaides, K. H. (2021). Ophthalmic artery Doppler in combination with other biomarkers in prediction of pre-eclampsia at 19-23 weeks' gestation. *Ultrasound Obstet Gynecol*, *57*(1), 75-83. <https://doi.org/10.1002/uog.23528>

Sarno, M., Wright, A., Vieira, N., Sapantzoglou, I., Charakida, M., & Nicolaides, K. H. (2021). Ophthalmic artery Doppler in combination with other biomarkers in prediction of pre-eclampsia at 35-37 weeks' gestation. *Ultrasound Obstet Gynecol*, *57*(4), 600-606. <https://doi.org/10.1002/uog.23517>

SB, E. H. M., Park, F., Murthi, P., Martins, W. P., Kane, S. C., Williams, P., Hyett, J., & da Silva Costa, F. (2016). TNF-R1 as a first trimester marker for prediction of pre-eclampsia. *J Matern Fetal Neonatal Med*, *29*(6), 897-903. <https://doi.org/10.3109/14767058.2015.1022865>

Scazzocchio, E., Crovetto, F., Triunfo, S., Gratacos, E., & Figueras, F. (2017). Validation of a first-trimester screening model for pre-eclampsia in an unselected population. *Ultrasound Obstet Gynecol*, *49*(2), 188-193. <https://doi.org/10.1002/uog.15982>

Scazzocchio, E., Figueras, F., Crispi, F., Meler, E., Masoller, N., Mula, R., & Gratacos, E. (2013). Performance of a first-trimester screening of preeclampsia in a routine care low-risk setting. *Am J Obstet Gynecol*, *208*(3), 203 e201-203 e210. <https://doi.org/10.1016/j.ajog.2012.12.016>

Skrastad, R. B., Hov, G. G., Blaas, H. G., Romundstad, P. R., & Salvesen, K. A. (2015). Risk assessment for preeclampsia in nulliparous women at 11-13 weeks gestational age: prospective evaluation of two algorithms. *Bjog*, *122*(13), 1781-1788. <https://doi.org/10.1111/1471-0528.13194>

Sonek, J., Krantz, D., Carmichael, J., Downing, C., Jessup, K., Haidar, Z., Ho, S., Hallahan, T., Kliman, H. J., & McKenna, D. (2018). First-trimester screening for early and late preeclampsia using maternal characteristics, biomarkers, and estimated placental volume. *Am J Obstet Gynecol*, *218*(1), 126 e121-126 e113. <https://doi.org/10.1016/j.ajog.2017.10.024>

Tan, M. Y., Syngelaki, A., Poon, L. C., Rolnik, D. L., O'Gorman, N., Delgado, J. L., Akolekar, R., Konstantinidou, L., Tsavdaridou, M., Galeva, S., Ajdacka, U., Molina, F. S., Persico, N., Jani, J. C., Plasencia, W., Greco, E., Papaioannou, G., Wright, A., Wright, D., & Nicolaides, K. H. (2018). Screening for pre-eclampsia by maternal factors and biomarkers at 11-13 weeks' gestation. *Ultrasound Obstet Gynecol*, *52*(2), 186-195. <https://doi.org/10.1002/uog.19112>

Tan, M. Y., Wright, D., Syngelaki, A., Akolekar, R., Cicero, S., Janga, D., Singh, M., Greco, E., Wright, A., Maclagan, K., Poon, L. C., & Nicolaides, K. H. (2018). Comparison of diagnostic accuracy of early screening for pre-eclampsia by NICE guidelines and a method combining maternal factors and biomarkers: results of SPREE. *Ultrasound Obstet Gynecol*, *51*(6), 743-750. <https://doi.org/10.1002/uog.19039>

Teixeira, C., Tejera, E., Martins, H., Pereira, A. T., Costa-Pereira, A., & Rebelo, I. (2014). First trimester aneuploidy screening program for preeclampsia prediction in a portuguese obstetric population. *Obstet Gynecol Int*, *2014*, 435037. <https://doi.org/10.1155/2014/435037>

Teoh, S. S. Y., Wang, Y., Li, Y., Leemaqz, S. Y., Dekker, G. A., Roberts, C. T., & Nie, G. (2019). Low Serum Levels of HtrA3 at 15 Weeks of Gestation Are Associated with Late-Onset Preeclampsia Development and Small for Gestational Age Birth. *Fetal Diagn Ther*, *46*(6), 392-401. <https://doi.org/10.1159/000497144>

Tsiakkas, A., Saiid, Y., Wright, A., Wright, D., & Nicolaides, K. H. (2016). Competing risks model in screening for preeclampsia by maternal factors and biomarkers at 30-34 weeks' gestation. *Am J Obstet Gynecol*, *215*(1), 87 e81-87 e17. <https://doi.org/10.1016/j.ajog.2016.02.016>

Valino, N., Giunta, G., Gallo, D. M., Akolekar, R., & Nicolaides, K. H. (2016a). Biophysical and biochemical markers at 30-34 weeks' gestation in the prediction of adverse perinatal outcome. *Ultrasound Obstet Gynecol*, *47*(2), 194-202. <https://doi.org/10.1002/uog.14928>

Valino, N., Giunta, G., Gallo, D. M., Akolekar, R., & Nicolaides, K. H. (2016b). Biophysical and biochemical markers at 35-37 weeks' gestation in the prediction of adverse perinatal outcome. *Ultrasound Obstet Gynecol*, *47*(2), 203-209. <https://doi.org/10.1002/uog.15663>

Zanello, M., Sekizawa, A., Purwosunu, Y., Curti, A., & Farina, A. (2014). Circulating mRNA for the PLAC1 gene as a second trimester marker (14-18 weeks' gestation) in the screening for late preeclampsia. *Fetal Diagn Ther*, *36*(3), 196-201. <https://doi.org/10.1159/000360854>

Zhang, J., Han, L., Li, W., Chen, Q., Lei, J., Long, M., Yang, W., Li, W., Zeng, L., & Zeng, S. (2019). Early prediction of preeclampsia and small-for-gestational-age via multi-marker model in Chinese pregnancies: a prospective screening study. *BMC Pregnancy Childbirth*, *19*(1), 304. <https://doi.org/10.1186/s12884-019-2455-8>
